# HLTF cooperates with GATA1 to activate transcriptional programs and chromatin remodeling during erythroid development

**DOI:** 10.1093/nar/gkaf1506

**Published:** 2026-01-09

**Authors:** Han Gong, Bin Hu, Ling Nie, Huifang Zhang, Pan Wang, Wenwen Xu, Maohua Li, Li Liu, Ji Zhang, Xiaojuan Xiao, Long Liang, Yongbin Chen, Mao Ye, Narla Mohandas, Hongling Peng, Xu Han, Yue Sheng, Jing Liu

**Affiliations:** Department of Hematology, The Second Xiangya Hospital, Molecular Biology Research Center, School of Life Sciences, Hunan Province Key Laboratory of Basic and Applied Hematology, Central South University, Hunan,China; Department of Hematology, The Second Xiangya Hospital, Molecular Biology Research Center, School of Life Sciences, Hunan Province Key Laboratory of Basic and Applied Hematology, Central South University, Hunan, China; School of Life and Environmental Sciences, Hangzhou Normal University, Hangzhou, China; Department of Hematology, Xiangya Hospital, Central South University, Hunan, China; Department of Hematology, The Second Xiangya Hospital, Molecular Biology Research Center, School of Life Sciences, Hunan Province Key Laboratory of Basic and Applied Hematology, Central South University, Hunan, China; Department of Hematology, The Second Xiangya Hospital, Molecular Biology Research Center, School of Life Sciences, Hunan Province Key Laboratory of Basic and Applied Hematology, Central South University, Hunan, China; Department of Hematology, The Second Xiangya Hospital, Molecular Biology Research Center, School of Life Sciences, Hunan Province Key Laboratory of Basic and Applied Hematology, Central South University, Hunan, China; Department of Hematology, The Second Xiangya Hospital, Molecular Biology Research Center, School of Life Sciences, Hunan Province Key Laboratory of Basic and Applied Hematology, Central South University, Hunan, China; Department of Hematology, The Second Xiangya Hospital, Molecular Biology Research Center, School of Life Sciences, Hunan Province Key Laboratory of Basic and Applied Hematology, Central South University, Hunan, China; The Affiliated Nanhua Hospital, Department of Clinical Laboratory, Hengyang Medical School, University of South China, Hunan, China; Department of Hematology, The Second Xiangya Hospital, Molecular Biology Research Center, School of Life Sciences, Hunan Province Key Laboratory of Basic and Applied Hematology, Central South University, Hunan, China; Department of Hematology, The Second Xiangya Hospital, Molecular Biology Research Center, School of Life Sciences, Hunan Province Key Laboratory of Basic and Applied Hematology, Central South University, Hunan, China; Key Laboratory of Animal Models and Human Disease Mechanisms of Chinese Academy of Sciences & Yunnan Province, Kunming Institute of Zoology, Yunnan, China; Molecular Science and Biomedicine Laboratory (MBL), State Key Laboratory of Chemo/Biosensing and Chemometrics, College of Biology, College of Chemistry and Chemical Engineering, Aptamer Engineering Center of Hunan Province, Hunan University, Hunan, China; Research Laboratory of Red Cell Physiology, New York Blood Center, New York, United States; Department of Hematology, The Second Xiangya Hospital, Molecular Biology Research Center, School of Life Sciences, Hunan Province Key Laboratory of Basic and Applied Hematology, Central South University, Hunan, China; Department of Hematology, The Second Xiangya Hospital, Molecular Biology Research Center, School of Life Sciences, Hunan Province Key Laboratory of Basic and Applied Hematology, Central South University, Hunan, China; Department of Hematology, The Second Xiangya Hospital, Molecular Biology Research Center, School of Life Sciences, Hunan Province Key Laboratory of Basic and Applied Hematology, Central South University, Hunan, China; Department of Hematology, The Second Xiangya Hospital, Molecular Biology Research Center, School of Life Sciences, Hunan Province Key Laboratory of Basic and Applied Hematology, Central South University, Hunan, China

## Abstract

Erythropoiesis, the process by which hematopoietic stem cells differentiate into mature red blood cells, is tightly regulated by a complex network of transcriptional and epigenetic mechanisms. While helicase-like transcription factor (HLTF) is known for its roles in transcriptional regulation, chromatin remodeling, and genome stability, its function in erythroid development has remained unexplored. Here, we identify HLTF as a novel regulator of GATA1 and erythropoiesis. HLTF directly binds the GATA1 promoter, enhancing its transcription. *In vitro* and *in vivo* assays demonstrated that HLTF promotes erythroid proliferation, survival, and terminal differentiation. HLTF knockout impairs erythropoiesis, effects which are partially rescued by GATA1 overexpression. Multi-omics analyses (RNA-seq, ATAC-seq, and CUT&Tag) reveal that HLTF maintains chromatin accessibility and activates erythroid gene networks. HLTF interacts with GATA1, co-occupies erythroid regulatory regions, and facilitates GATA1 genomic binding. Notably, GATA1 also transcriptionally activates HLTF, forming a positive feedback loop. In erythroid disorders, HLTF is up-regulated in polycythemia vera (PV) and down-regulated in myelodysplastic syndromes (MDS). Knockout of HLTF in PV patient-derived cells suppressed erythroid hyperplasia, reduced chromatin accessibility, and impaired GATA1 binding. Together, these findings reveal HLTF as a transcriptional regulator of GATA1 and a pivotal modulator of erythropoiesis, providing new insights into erythroid lineage control and erythroid-related diseases.

## Graphical Abstract

**Graphical Abstract:**
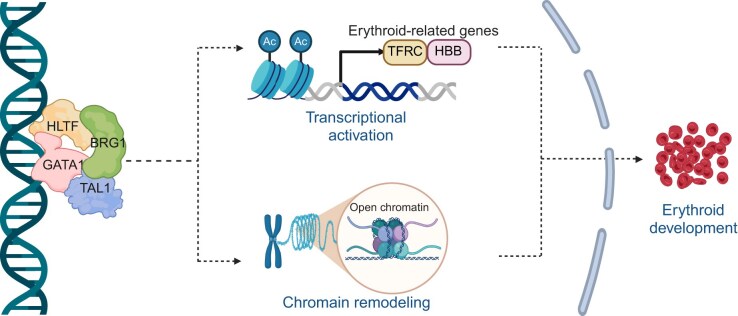

## Introduction

In humans, ~2 million red blood cells (RBCs) are produced each second to meet physiological demands [1]. This process, known as erythropoiesis, involves the proliferation and differentiation of hematopoietic stem cells (HSCs) into mature RBCs and occurs in three primary stages: early progenitor proliferation, terminal erythroid differentiation, and reticulocyte maturation [2]. Terminal erythroid differentiation constitutes a fundamentally distinct biological process, characterized not by proliferative cycling but by tightly regulated stages of differentiation culminating in chromatin condensation and enucleation [3, 4]. A central regulator of erythropoiesis is the transcription factor (TF) GATA-binding factor 1 (GATA1), which directly controls the expression of numerous erythroid-specific genes and is essential for the survival and terminal differentiation of erythroid progenitors [5, 6]. GATA1 expression and transcriptional activity are dynamically regulated during erythroid differentiation, and its dysregulation can lead to a range of hematological disorders, including polycythemia vera (PV) and myelodysplastic syndromes (MDS) [7, 8]. Several proteins, including USP7, HSP70, and CDKN2D, modulate GATA1 protein stability [9–11]. Notably, GATA1 also autoregulates its own transcription [12]. Additional upstream regulators of GATA1 transcription have been identified, including Meis1 and Hi1α in zebrafish primitive erythropoiesis [13, 14], and the transcriptional cofactor HES6 in human hematopoiesis [15]. However, the upstream transcriptional regulation of GATA1—especially in humans and in hematological disorders—remains incompletely understood.

Leveraging biotin-labeled GATA1 promoter pull-down assays coupled with mass spectrometry (MS), helicase-like transcription factor (HLTF) was identified as an upstream transcriptional regulator of GATA1. HLTF, also known as SMARCA3, is a member of the SWI/SNF family of ATP-dependent chromatin remodelers and was initially identified based on its homology to the yeast Rad5 protein [16, 17]. HLTF is a multifunctional protein with established roles in maintaining genome integrity [18, 19], suppressing G-quadruplex (G4) structures [20], functioning as a ubiquitin ligase for H3K23 in cancer [21] and a transcriptional activator of oncogenes [22, 23], and modulating chromatin accessibility in circadian regulation [24]. HLTF has been reported to transcriptionally activate β-globin expression in K562 cells [25]. Despite its broad functional repertoire, HLTF’s role in hematopoiesis, particularly in erythroid development, has not been elucidated.

In this study, we used DNA pull-down combined with MS technology to identify HLTF as a potential upstream regulatory factor of GATA1. Comprehensive analyses using assay for transposase-accessible chromatin with high-throughput sequencing (ATAC-seq), cleavage under targets and tagmentation (CUT&Tag), and RNA-sequencing (RNA-seq) identified HLTF downstream targets and regulatory pathways in erythroid cells. Furthermore, we validated HLTF’s expression pattern in patient-derived samples: HLTF was silenced in samples from patients with PV and overexpressed in samples from patients with MDS, revealing its impact on erythroid differentiation. Together, these findings uncover a previously unrecognized role and mechanism of HLTF in erythropoiesis and provide new insights into the molecular control of erythropoiesis and erythroid-related disorders.

## Materials and methods

### Antibodies and reagents

All antibodies and reagents used for flow cytometry and western blot analyses are listed in Supplementary Table S1. All primers, single guide RNAs (sgRNAs), and short hairpin RNAs (shRNAs) are listed in Supplementart Table S2.

### DNA pull-down assay

DNA pull-down assays were performed to identify potential GATA1 promoter-binding proteins. Biotinylated DNA probes corresponding to the GATA1 promoter region (Supplementary Table S3) were synthesized and used for the pull-down assays.

To study the binding of HLTF to the GATA1 promoter, the JASPA database [26] was used to predict the core binding sequence of HLTF to the GATA1 promoter. Wild-type (WT) and motif-mutated DNA probes were subsequently designed and used for DNA pull-down assays.

### Chromatin immunoprecipitation-quantitative PCR

Chromatin immunoprecipitation (ChIP) assays were performed to assess the binding of HLTF to the GATA1 promoter region in erythroid cells. Briefly, day 9 (D9) erythroid cells were cross-linked with 1% formaldehyde for 10 min at room temperature to preserve protein–DNA interactions. The cross-linking reaction was quenched by adding 0.125 M glycine, followed by washing the cells twice with ice-cold phosphate-buffered saline (PBS). The cells were then lysed in ChIP lysis buffer [50 mM Tris–HCl, pH 8.0, 10 mM EDTA, 1% sodium dodecylsulfate (SDS), 1 mM dithiothreitol (DTT), and protease inhibitors], and chromatin was sheared to an average size of 200–500 bp using a Bioruptor Sonicator (Diagenode, Liège, Belgium). Sheared chromatin was incubated overnight at 4°C with 2 µg of anti-HLTF antibody or control IgG (Cell Signaling Technology). Protein-A/G Sepharose beads (Sigma-Aldrich) were then added to the chromatin–antibody complex, and the mixture was incubated for an additional 4 h at 4°C with gentle rotation to capture the immunocomplexes. After washing the beads with low salt, high salt, and LiCl buffers, the cross-links were reversed by incubation at 65°C overnight. The chromatin was then purified using a PCR purification kit (Qiagen, Hilden, Germany).

To assess HLTF binding to the GATA1 promoter, purified chromatin was analyzed by quantitative PCR (qPCR). A standard qPCR protocol was used, with the qPCR performed using SYBR Green Master Mix (Thermo Fisher Scientific) and a QuantStudio 5 Real-Time PCR System (Thermo Fisher Scientific). Data were normalized to the input chromatin and the non-specific binding control (IgG). The relative enrichment of HLTF binding at the GATA1 promoter was calculated using the ΔΔCt method.

### Erythroid differentiation analysis

Erythroid differentiation was assessed by staining for erythroid markers, including CD235a (glycophorin A) and CD71 (transferrin receptor). Cells were incubated with fluorescence-conjugated monoclonal antibodies against CD71 and CD235a (BD) for 30 min at 4°C. After washing with PBS, the samples were analyzed by flow cytometry. The percentage of positive cells for CD235a and CD71 was used to determine the progression of erythroid differentiation at different stages (early, late erythroid, and reticulocyte).

### Bone marrow transplantation

To evaluate the *in vivo* role of HLTF in erythropoiesis, a bone marrow transplantation (BMT) assay was performed using HLTF knockdown (shHLTF) and control (shNC) mice. BMT was performed using c-Kit^+^ hematopoietic stem and progenitor cells (HSPCs) isolated from WT mice.

### RNA sequencing

Total RNA was extracted from HLTF knockout (KO) and control erythroid cells using the RNeasy Mini Kit (Qiagen) according to the manufacturer’s instructions. RNA quality and integrity were assessed using a NanoDrop spectrophotometer (Thermo Fisher Scientific) and by electrophoresis on a 1% agarose gel. RNA samples with a 260/280 nm absorbance ratio between 1.8 and 2.0 and an RNA integrity number (RIN) >7 were used for sequencing.

RNA-seq was performed using the Illumina HiSeq 4000 platform (Illumina), generating paired-end 150 bp reads. Raw sequencing data were processed and analyzed using the established bioinformatics pipeline. In brief, quality control (QC) of the raw sequencing data was performed using FastQC (http://www.bioinformatics.babraham.ac.uk/projects/fastqc/). Reads were then aligned to the human reference genome (GRCh38) using STAR aligner (version 2.7.3a). The resulting BAM files were used for gene expression analysis, with read counts determined using featureCounts (v1.6.4).

### ATAC-sequencing

ATAC-seq was performed to assess chromatin accessibility in HLTF-deficient and control cells during erythroid differentiation. The assay was carried out following the published protocol [27]. Sequencing was performed on an Illumina platform (Illumina HiSeq 4000) to generate paired-end 150 bp reads. Raw FASTQ files were processed for QC using FastQC (http://www.bioinformatics.babraham.ac.uk/projects/fastqc/) and trimmed to remove low-quality bases and adapter sequences. Reads were then aligned to the human reference genome (GRCh38) using Bowtie2 (version 2.3.4), and peak calling was performed using MACS2 (version 2.1.1) with default parameters to identify regions of accessible chromatin. Peaks were annotated using the ChIPseeker package [28] to identify genomic features associated with each peak.

### Cleavage under targets and tagmentation

CUT&Tag was used to investigate the binding of HLTF and GATA1 to their target regions in erythroid cells. The assay was performed as previously described [29] with modifications specific to our study.

### Ethics approval and consent to participate

The study adhered to the ethical principles laid out in the Declaration of Helsinki. All the samples of human healthy donors, and of MDS (diagnosed with refractory anemia with ringed sideroblasts) and PV (diagnosed with JAK2 mutation) patients used in the study were approved as per the ethical standards of the Ethics Committee of the School of Life Sciences of Central South University (approval no. 2019-1-11) and processed in accordance with the approved procedure of the committee. Written informed consent was obtained from all participants. The Animal Care and Use Committee of the Second Xiangya Hospital granted approval for all animal experiments (approval no. 2022647).

## Results

### HLTF transcriptionally regulates GATA1 and is essential for erythropoiesis

**Figure 1.**
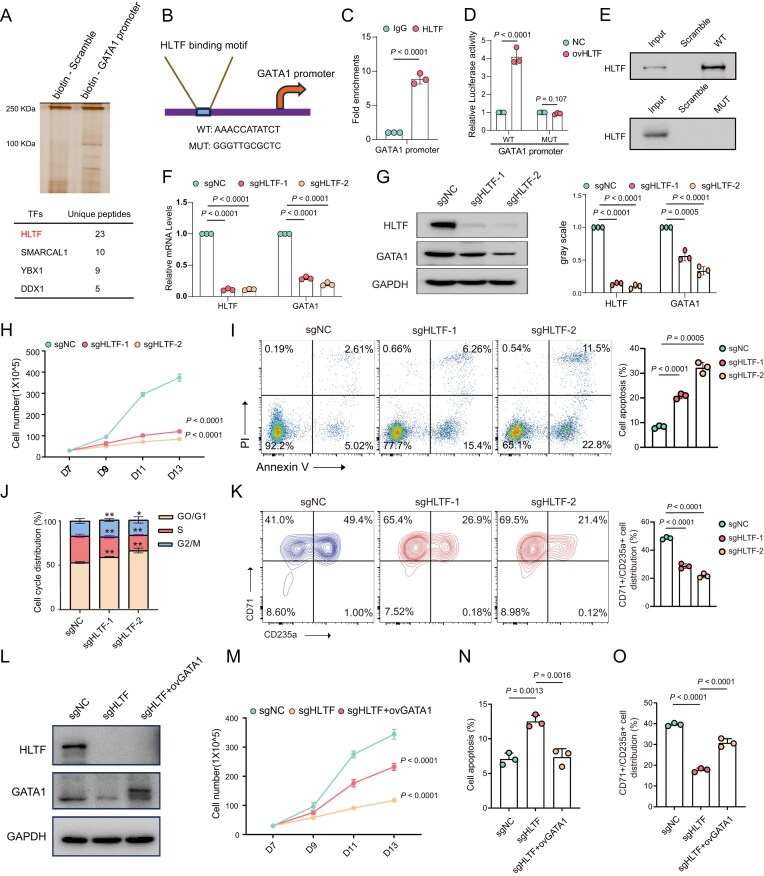
HLTF transcriptionally regulates GATA1 and is essential for erythropoiesis. (**A**) Schematic of the DNA pull-down assay using the biotin-labeled GATA1 promoter or scrambled control, followed by MS in erythroblast cells. Upper: silver staining of pulled-down proteins. Lower: TF candidate list. (**B**) Predicted HLTF-binding site within the GATA1 promoter (WT: AAACCATATCT) and its corresponding mutated sequence (MUT: GGGTTGCGCTC) based on the JASPAR database. (**C**) ChIP-qPCR confirms binding of HLTF to the WT core GATA1 promoter sequence in CD34^+^-derived erythroblast cells. (**D**) Dual-luciferase reporter assay using WT and mutated GATA1 promoter constructs co-transfected with the HLTF expression vector or IgG control. (**E**) DNA pull-down followed by western blot demonstrating that HLTF protein specifically interacts with the WT GATA1 promoter but not the mutant. (**F**) Relative mRNA levels of HLTF and GATA1 by qPCR in CD34^+^-derived erythroblast cells. (**G**) Western blot results of HLTF and GATA1 protein levels in erythroblast cells. Glyceraldehyde-3-phosphate dehydrogenase (GAPDH) serves as a loading control. Right: quantification of HLTF and GATA1 protein levels from western blot. (**H**) Cell proliferation curves showing total cell numbers during erythroid differentiation from day 7 to day 13 (D7–D13). (**I**) Annexin V/propidium iodide (PI) staining followed by flow cytometry reveals increased apoptosis in HLTF-deficient cells compared with the sgNC control. (**J**) Cell cycle analysis in HLTF-deficient erythroblast cells. (**K**) Flow cytometric analysis of CD71^+^CD235a^+^ erythroid populations. (**L**) Western blot analysis of HLTF and GATA1 protein levels in sgNC, sgHLTF, and sgHLTF + ovGATA1 groups in CD34^+^-derived erythroblast cells. GAPDH serves as a loading control. (**M**) Cell proliferation curves. (**N**) Annexin V/PI staining showing that GATA1 overexpression partially rescues apoptosis induced by HLTF knockout. (**O**) Flow cytometric analysis of CD71^+^CD235a^+^ cells after transduction with sgNC, sgHLTF, or sgHLTF + ovGATA1. Data are representative of at least three independent experiments. Error bars indicate the mean ± standard deviation (SD).

To identify novel TFs that regulate GATA1, we performed DNA pull-down assays using a biotin-labeled GATA1 promoter in CD34^+^-derived erythroid cells. Following DNA pull-down, protein complexes were analyzed by MS. Among the proteins identified, HLTF was the most abundant TF candidate (Fig. 1A; Supplementary Table S4). Public bulk RNA-seq and single-cell RNA-seq [30] analyses suggested that HLTF is highly expressed in late-erythroid cells (Supplementary Fig. S1A, B). Furthermore, correlation analysis of sorted human erythroid cells [31, 32] demonstrated a strong positive correlation between HLTF and GATA1 at both mRNA and protein levels (Supplementary Fig. S1C, D). To precisely identify the binding site of HLTF on the GATA1 promoter, we used the JASPAR database to predict its core binding motif (Fig. 1B). ChIP-qPCR confirmed HLTF binding to the WT GATA1 promoter sequence in CD34^+^-derived erythroid cells (Fig. 1C). Luciferase reporter and DNA pull-down assays further confirmed that HLTF binds to the WT sequence but not to the mutant variant (Fig. 1D, E).

To investigate the functional significance of HLTF binding, we performed CRISPR/Cas9 [clustered regularly interspaced palindromic repeats (CRISPR)/CRISPR-associated protein 9]-mediated knockout of HLTF in CD34^+^-derived erythroid cells. Successful genome editing at the HLTF locus was confirmed by Sanger sequencing (Supplementary Fig. S1E, F). qPCR and western blot analyses revealed that GATA1 mRNA and protein levels were significantly down-regulated in HLTF-deficient cells, indicating that HLTF transcriptionally regulates GATA1 expression (Fig. 1F, G). Functionally, HLTF depletion significantly impaired cell proliferation, increased apoptosis, induced cell cycle arrest, and delayed erythroid differentiation (Fig. 1H–K). Similar results were observed in human umbilical cord-derived progenitor erythroid 2 (HUDEP-2) cells (Supplementary Fig. S2A–E). To assess whether these phenotypes were mediated through GATA1, we overexpressed GATA1 in HLTF-deficient cells (Fig. 1L). Rescue experiments showed that GATA1 overexpression partially reversed the proliferation defects, apoptosis, and differentiation delays caused by HLTF loss (Fig. 1M–O).

Collectively, these results establish HLTF as a novel transcriptional regulator essential for GATA1 expression during erythropoiesis.

### Knockdown of HLTF impaired erythroid development *in vivo*

**Figure 2.**
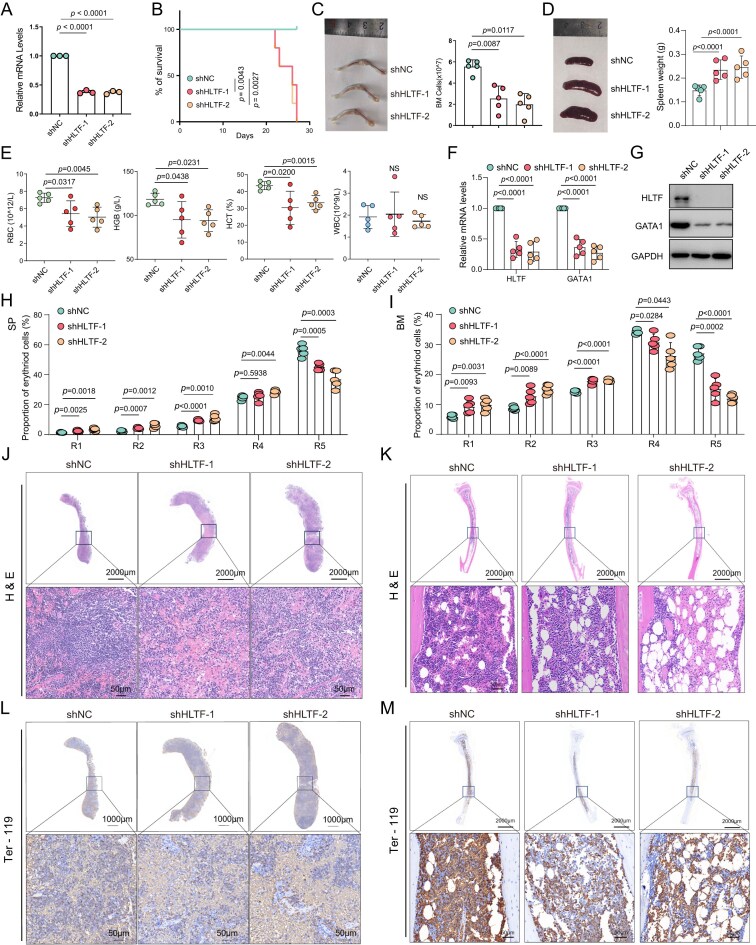
Hltf knockdown *in vivo* resulted in reduced Gata1 expression and impaired erythropoiesis. (**A**) qPCR analysis of Hltf in the mouse bone marrow *in vitro* culture experiment. (**B**) Kaplan–Meier survival analysis indicated that Hltf knockdown led to significantly shortened survival in recipient mice. (**C**) Representative images of mouse leg bones. Total bone marrow cell counts were found to be reduced in shHltf mice compared with controls. (**D**) Representative images and statistics of mouse spleen. (**E**) Hematological analysis reveals reduced RBC counts, HGB levels, HCT levels, and WBC counts in shHltf mice. (**F**) qPCR results suggesting that Hltf knockdown significantly decreased the mRNA levels of both Hltf and Gata1. (**G**) Western blot results of Hltf and Gata1 protein levels in bone marrow cells transduced with shNC or shHltf constructs. GAPDH serves as a loading control. (**H** and **I**) Flow cytometry showed decreased proportions of mature erythroid cells in both the spleen (H) and bone marrow (I) following Hltf knockdown. (**J** and **K**) Hematoxylin and eosin (H&E) staining demonstrated hypocellularity in both spleen (J) and bone marrow (K) tissues of knockdown mice. (**L** and **M**) Immunohistochemistry for Ter-119 revealed increased Ter-119^+^ cells in spleen (L) and decreased erythroid cell staining in bone marrow (M). Data are representative of at least five biological replicates. Error bars indicate the mean ± SD. NS: not significant.

To further investigate HLTF’s function *in vivo*, we performed a BMT assay using Hltf shRNA or control shRNA lentivirus. c-kit^+^ HSPCs were isolated from WT mice and then transplanted into lethally irradiated recipient mice. Prior to the main experiments, two shRNA sequences were screened and validated for their efficiency in reducing Hltf expression (Fig. 2A). Subsequently, two independent transplantation cohorts were established—one for survival analysis and the other for phenotypic evaluation. Kaplan–Meier analysis revealed that Hltf knockdown significantly reduced survival compared with controls (Fig. 2B). In the phenotypic cohort, upon sacrifice, mice receiving Hltf-deficient cells displayed visibly pale bones and reduced bone marrow cellularity (Fig. 2C), while spleen size and weight were markedly increased (Fig. 2D). Complete blood count analysis further revealed that Hltf knockdown resulted in significantly reduced RBC counts, hemoglobin (HGB) levels, and hematocrit (HCT) levels, whereas white blood cell (WBC) counts remained unchanged (Fig. 2E). Both qPCR and western blot analyses confirmed that the two independent shRNAs effectively suppressed HLTF expression and concurrently down-regulated its downstream target GATA1 (Fig. 2F, G).

Terminal erythropoiesis in the spleen and bone marrow was assessed by flow cytometry following previously established protocols [3]. Compared with the control group, Hltf knockdown mice showed an increased proportion of proerythroblasts and a reduction in mature RBCs in both the bone marrow and spleen (Fig. 2H, I; Supplementary Fig. S3A, B).

Hematoxylin and eosin (H&E) staining of spleen sections revealed splenomegaly with dilated and architectural abnormalities (Fig. 2J). H&E analysis of bone marrow sections indicated decreased overall cellularity and prominent adipocyte vacuolation in the HLTF-deficient group (Fig. 2K). Immunohistochemical staining showed increased extramedullary hematopoiesis in the spleen of Hltf knockdown mice, while the number of Ter-119^+^ erythroid cells was reduced in the bone marrow (Fig. 2L–M).

We next evaluated the functional impact of HLTF loss on hematopoietic progenitor activity using a colony-forming unit (CFU) assay. Bone marrow cells from Hltf-deficient and control mice were cultured in methylcellulose medium, and colony numbers were quantified after 10 days. Hltf knockdown significantly decreased the numbers of CFU-GEMM, CFU-GM, and BFU-E colonies compared with controls (Supplementary Fig. S3C, D).

Together, these results indicate that HLTF is critical for maintaining normal erythropoiesis *in vivo*. Loss of HLTF leads to reduced GATA1 expression, impaired RBC maturation, anemia, and compensatory extramedullary hematopoiesis.

### HLTF deficiency leads to decreased chromatin accessibility and impaired erythroid transcriptionally programs

**Figure 3.**
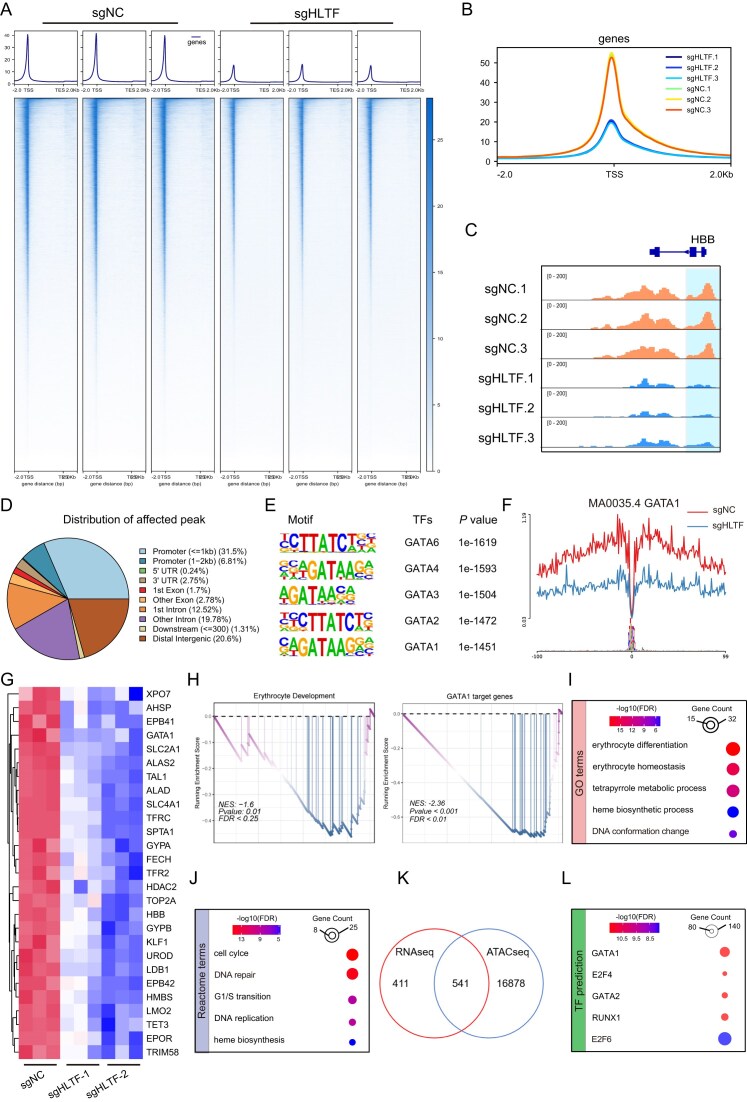
HLTF deficiency leads to down-regulation of GATA1 target genes and erythroid gene signatures. (**A**) Metagene plot showing chromatin accessibility profiles across gene bodies in HLTF KO versus control erythroblast cells at D9. (**B**) Aggregated accessibility plot centered on TSSs, demonstrating decreased accessibility in HLTF-deficient cells. (**C**) Genome browser tracks at β-globin promoter regions. (**D**) Genomic features of the affected peak. (**E**) Motif analysis of the affected peak. (**F**) Chromatin accessibility analysis at GATA1-bound erythroid genes suggesting significant reduction in GATA1 binding ability upon HLTF loss. (**G**) Heatmap displaying expression patterns of representative erythroid-related genes across HLTF KO and control samples. Red indicates higher gene expression levels, and blue indicates lower gene expression levels. (**H**) Gene set enrichment analysis (GSEA) results. (**I** and **J**) Enrichment results of erythropoiesis-related biological processes based on the Gene Ontology (GO) (I) and Reactome (J) databases. (**K**) Venn diagram showing overlap between differentially expressed genes from RNA-seq and genes associated with differential ATAC-seq peaks. (**L**) TF motif enrichment analysis identifying putative upstream regulators of the intersecting genes. NES, normalized enrichment score; FDR, false discovery rate.

Given HLTF’s known role in chromatin remodeling [24], we performed ATAC-seq to comprehensively investigate the role of HLTF in erythropoiesis. This analysis revealed a global reduction in accessible chromatin regions in HLTF-deficient erythroid cells relative to controls (Fig. 3A). The majority of differential accessibility peaks were located within ± 2 kb of transcription start sites (TSSs; Fig. 3B). Correlation analysis further confirmed distinct chromatin accessibility profiles between HLTF-deficient and control cells (Supplementary Fig. S4A), and differential peak analysis identified 17 419 regions with altered accessibility—most of which were down-regulated in HLTF KO cells (Supplementary Fig. S4B). Notably, IGV browser visualization showed significantly reduced chromatin accessibility at the promoter region of the erythroid marker gene HBB in HLTF-deficient cells (Fig. 3C), whereas the chromatin accessibility at the promoter region of the positive control gene SMARCA4 was not significantly altered (Supplementary Fig. S4C). Structural analysis revealed that ~40% of affected peaks were localized to promoter regions (Fig. 3D). Motif analysis showed decreased accessibility at GATA motifs (Fig. 3E), and footprinting analysis using the HINT-ATAC algorithm [33] revealed reduced ATAC accessibility at regulatory elements harboring HLTF- and GATA1-binding sites in HLTF-deficient cells (Fig. 3F; Supplementary Fig. S4D).

To further investigate the effect of HLTF on gene expression, we conducted RNA-seq in HLTF-deficient and control erythroid cells. Notably, both GATA1 and its well-characterized downstream targets were significantly down-regulated in HLTF-deficient cells, as shown by volcano plots and heatmaps (Fig. 3G; Supplementary Fig. S4E, F). Gene set enrichment analysis (GSEA) revealed significant negative enrichment of gene sets related to erythrocyte development, GATA1 targets, and HGB binding in the HLTF-deficient cells (Fig. 3H; Supplementary Fig. S4G). Furthermore, GO and Reactome enrichment analyses of differentially expressed genes identified strong associations with key erythroid processes, including erythrocyte differentiation, erythrocyte homeostasis, heme metabolism, and heme biosynthesis (Fig. 3I, J; Supplementary Fig. S4H).

Integrative analysis of RNA-seq and ATAC-seq data identified 541 genes whose differential expression correlated with altered chromatin accessibility (Fig. 3K). TF enrichment analysis showed that a significant proportion of these genes were known GATA1 targets (Fig. 3L), highlighting the interdependence of HLTF and GATA1 in orchestrating the erythroid transcriptional program.

Together, these results demonstrate that HLTF is essential for maintaining chromatin accessibility and transcriptional activation of GATA1 and its downstream erythroid gene network. Loss of HLTF disrupts this regulatory axis, impairing erythroid maturation at both the epigenetic and transcriptional levels.

### HLTF cooperates with GATA1 to activate erythroid transcriptional programs

**Figure 4.**
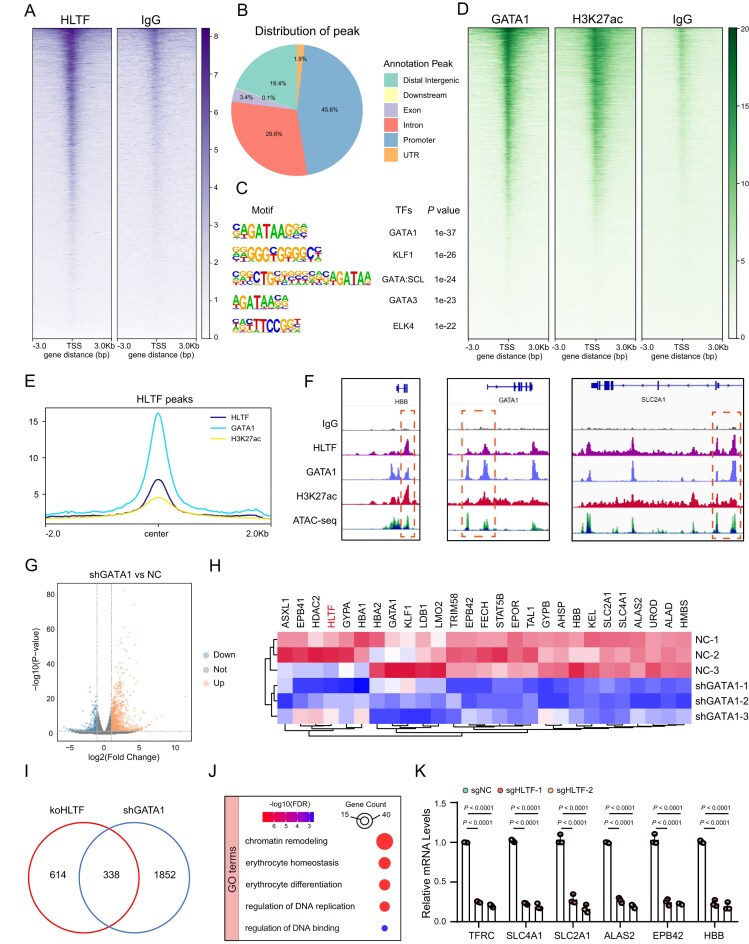
HLTF and GATA1 co-occupy erythroid regulatory regions and co-regulate downstream targets. (**A**) Heatmap of CUT&Tag profiling showing HLTF enrichment relative to the IgG control across genomic promoter regions in CD34^+^-derived erythroblast cells. (**B**) Pie chart illustrating the genomic distribution of HLTF-binding peaks, categorized by their proximity to genomic features [distal intergenic, downstream, exon, intron, promoter, and untranslated region (UTR)]. (**C**) Enriched TF-binding motifs identified within HLTF-bound regions, along with their corresponding TFs and *P*-values. (**D**) Heatmaps of CUT&Tag signals showing GATA1, H3K27ac, and IgG enrichment across genomic promoter regions. (**E**) Average profile of HLTF, GATA1, and H3K27ac CUT&Tag signals centered at HLTF-bound peaks. (**F**) Genome browser tracks displaying CUT&Tag signals for HLTF, GATA1, and H3K27ac, and ATAC-seq for representative erythroid differentiation genes such as HBB, GATA1, and SLC2A1. (**G**) Volcano plot illustrating differentially expressed genes after GATA1 knockdown in erythroid cells at D9. (**H**) Heatmap illustrating the expression levels of selected erythroid differentiation-related genes in CD34^+^-derived erythroblast cells treated with NC or shGATA1. Red indicates higher gene expression levels, and blue indicates lower gene expression levels. (**I**) Venn diagram illustrating the overlap between genes affected by HLTF knockout (sgHLTF) and GATA1 knockdown (shGATA1). (**J**) GO enrichment analysis of co-regulated targets. (**K**) Relative mRNA levels of key erythroid differentiation genes (TFRC, SLC4A1, SLC2A1, ALAS2, EPB42, and HBB) in sgNC-, sgHLTF-1-, and sgHLTF-2-treated erythroid cells. Data represent findings from at least three independent experiments. Error bars indicate the mean ± SD.

To investigate how HLTF regulates gene expression at the genomic level, we performed CUT&Tag using an HLTF-specific antibody in D9 erythroid cells. Genome-wide profiling revealed that HLTF-binding sites were predominantly enriched in promoter, intronic, and distal intergenic regions (Fig. 4A, B). Interestingly, motif analysis of HLTF-bound sites showed strong enrichment for erythroid TF motifs, particularly GATA1 and KLF1 (Fig. 4C). To support these findings, HLTF ChIP-seq data from the ENCODE project [34] were analyzed. Signal distribution showed strong concordance with CUT&Tag HLTF peaks observed in erythroblasts (Supplementary Fig. S5A). Genome annotation revealed that 54.8% of HLTF-binding peaks were located within promoter regions (Supplementary Fig. S5B). Consistent with CUT&Tag results, motif analysis confirmed that the GATA1-binding motif was among the most significantly enriched (Supplementary Fig. S5C). Functional enrichment analysis of HLTF target genes identified through peak annotation further supported their strong association with erythroid-related biological processes, including erythrocyte differentiation and heme biosynthesis (Supplementary Fig. S5D). These findings suggest that HLTF may function cooperatively with GATA1.

To examine this interaction more closely, we conducted additional CUT&Tag profiling for GATA1 and the active chromatin mark H3K27ac. Heatmap analysis showed strong GATA1 and H3K27ac binding at promoter regions across the genome (Fig. 4D). Co-localization analysis demonstrated substantial overlap among HLTF, GATA1, and H3K27ac signals at HLTF-bound regions (Fig. 4E). IGV browser visualization confirmed this co-occupancy at the promoters of representative erythroid genes, including HBB, GATA1, and SLC2A1 (Fig. 4F).

To identify shared downstream targets, we performed RNA-seq following GATA1 knockdown in CD34^+^-derived erythroid cells. Differential expression analysis revealed 791 up-regulated and 1372 down-regulated genes (Fig. 4G, H), the majority of which corresponded to known GATA1 targets. By intersecting the target of HLTF and GATA1, we identified 338 genes co-regulated by HLTF and GATA1 (Fig. 4I; Supplementary Table S5). Functional enrichment analysis of these shared targets revealed significant involvement in pathways such as chromatin remodeling, erythroid differentiation, and DNA replication (Fig. 4J). To validate these findings, we selected representative GATA1 targets (including TFRC and SLC4A1) for qPCR analysis. The results showed that HLTF knockout significantly reduced the mRNA expression of these genes (Fig. 4K).

To minimize potential effects of cell subpopulation variability, we knocked down HLTF or GATA1 in erythroblasts and used flow cytometry to sort CD71^+^CD235a^+^ cell populations for bulk RNA-seq (Supplementary Fig. S6A). Principal component analysis (PCA) and correlation analyses confirmed that HLTF and GATA1 knockdown samples exhibited similar transcriptional profiles that were clearly distinct from controls (Supplementary Fig. S6B, C). Heatmap analysis and GSEA showed that both knockdowns led to significant down-regulation of erythroid-related and GATA1 target genes (Supplementary Fig. S6D–F). Together, these findings confirm the consistency of our omics results and demonstrate that the observed effects are not attributable to differences in cell population composition.

Together, these results indicate that HLTF functionally cooperates with GATA1 to co-activate critical transcriptional programs that govern erythroid development.

### GATA1 directly regulates HLTF and establishes a positive feedback loop

**Figure 5.**
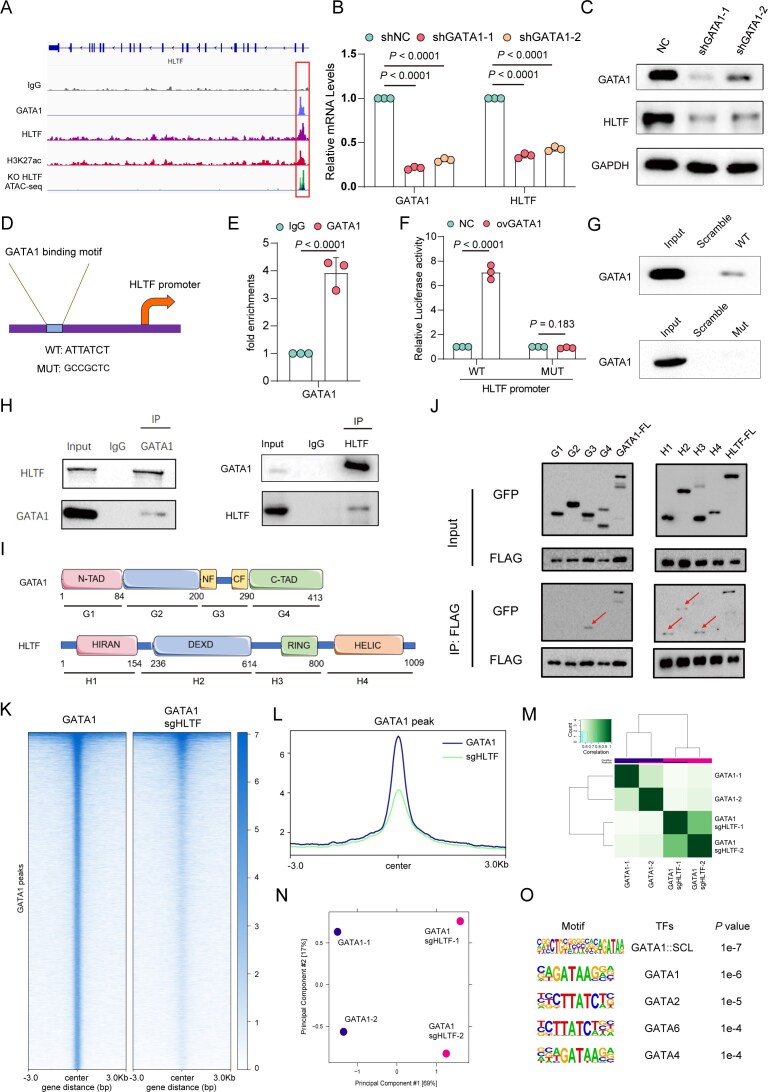
GATA1 was shown to directly activate HLTF transcription, forming a reciprocal regulatory loop. (**A**) IGV browser tracks at the HLTF locus, suggesting that GATA1 was enriched at the HLTF promoter region, with co-localized signals from H3K27ac and decreased ATAC-seq signal in HLTF KO (dark blue) cells compared with WT (light green) cells. (**B**) Relative mRNA levels of GATA1 and HLTF in in CD34^+^-derived erythroblast cells transduced with shNC (non-targeting control), shGATA1-1, or shGATA1-2. (**C**) Western blot analysis of GATA1 and HLTF protein levels in cells transduced with shNC, shGATA1-1, or shGATA1-2. GAPDH was used as a loading control. (**D**) Schematic representation of the GATA1-binding motif within the HLTF promoter, showing both the WT (ATTATCT) and mutated (MUT: GCCGCTC) sequences. (**E**) ChIP-qPCR analysis showing GATA1 enrichment at the HLTF promoter region, comparing IgG control and GATA1 immunoprecipitation in CD34^+^-derived erythroblast cells. (**F**) Luciferase reporter assay showing the relative luciferase activity of the HLTF promoter (WT or MUT) in cells co-transfected with NC (negative control) or ovGATA1. (**G**) DNA pull-down assays suggesting GATA1 enrichment at the HLTF promoter in WT but not MUT constructs. (**H**) Co-immunoprecipitation (Co-IP) experiments showing the interaction between GATA1 and HLTF. Left panel: IP with anti-GATA1 antibody, blotting for HLTF and GATA1. Right panel: IP with anti-HLTF antibody, blotting for GATA1 and HLTF. (**I**) Schematic diagram of the protein domains of HLTF and GATA1. (**J**) Domain mapping using truncated constructs revealing that the HIRAN, DEXD, and RING domains of HLTF interacted with the NF-CF region of GATA1. (**K**) CUT&Tag profiling of GATA1 after HLTF knockout on the GATA1-bound peak. (**L**) Average profile plot of the GATA1 CUT&Tag signal at GATA1 peaks in GATA1 and GATA1 sgHLTF cells. (**M**) The heatmap illustrating the correlation between GATA1 CUT&Tag profiles in control and HLTF-knockout samples . (**N**) The PCA plot. (**O**) Motif analysis of the diminished GATA1-bound peaks. Data are representative of at least three independent experiments. Error bars indicate the mean ± SD.

Bulk RNA-seq data showed that HLTF was decreased in GATA1 knockdown cells compared with the control cells (Fig. 4H), which indicated that HLTF may be regulated by GATA1. To investigate this possibility, we first examined the HLTF promoter for potential GATA1-binding motifs. CUT&Tag profiling demonstrated robust GATA1 enrichment at the HLTF promoter (Fig. 5A), and co-localization with ATAC-seq and H3K27ac signals further confirmed that this region exhibits open chromatin and active transcriptional features. Consistently, knockdown of GATA1 resulted in a marked decrease in HLTF mRNA and protein levels in CD34^+^-derived erythroid cells (Fig. 5B, C). ChIP-qPCR assays suggested significant enrichment at the predicted binding site (Fig. 5D, E). To test the functional relevance of this binding, we performed luciferase reporter assays. Overexpression of GATA1 significantly enhanced the transcriptional activity of the WT HLTF promoter, whereas mutation of the GATA1-binding motif abolished this effect (Fig. 5F). In agreement with this, DNA pull-down assays showed that GATA1 specifically binds the WT HLTF promoter probe, but not the mutated version (Fig. 5G).

We next explored whether HLTF and GATA1 physically interact. Co-immunoprecipitation (Co-IP) assays demonstrated the interaction between the two proteins in CD34^+^-derived erythroid cells (Fig. 5H). To dissect the domains involved, we performed interaction mapping using truncation constructs. The results revealed that the HIRAN, DEXD, and RING domains of HLTF interact with the NF-CF domain of GATA1 (Fig. 5I, J). Given that TAL1 and BRG1 are established GATA1 cofactors involved in chromatin organization and transcriptional regulation [35–37], we next examined whether HLTF participates in this complex. Co-IP analysis in erythroblasts demonstrated that HLTF associates with GATA1, TAL1, and BRG1 (Supplementary Fig. S7A). Moreover, HLTF knockdown weakened the interaction between GATA1 and both TAL1 and BRG1, suggesting that HLTF facilitates the assembly or stability of the TAL1–GATA1–BRG1 transcriptional complex (Supplementary Fig. S7B).

To assess whether HLTF influences GATA1’s chromatin binding capacity, we performed CUT&Tag profiling of GATA1 in HLTF-deficient erythroid cells. Notably, HLTF knockout significantly impaired GATA1 occupancy at promoter regions genome-wide (Fig. 5K, L). Correlation analysis and PCA further revealed a global shift in the GATA1 binding landscape following HLTF deletion (Fig. 5M, N). Motif analysis showed that the peaks most affected were enriched for canonical GATA motifs, suggesting reduced GATA1 engagement with its target elements in the absence of HLTF (Fig. 5O).

To further determine whether HLTF directly cooperates with GATA1 on chromatin, we performed an electrophoretic mobility shift assay (EMSA) using purified HLTF and GATA1 proteins and a biotin-labeled probe containing the GATA1-binding site. Incubation of the probe with either GATA1 or HLTF alone produced a single shifted band, while the combination of both proteins yielded three distinct shifted bands, corresponding to HLTF–DNA, GATA1–DNA, and HLTF–GATA1–DNA complexes (Supplementary Fig. S7C). The addition of a 25× unlabeled (“cold”) competitor probe markedly reduced the intensity of all shifted bands, confirming binding specificity. These findings demonstrate that HLTF and GATA1 can form a ternary complex on the same DNA fragment, supporting a model in which HLTF directly facilitates GATA1 binding to shared promoter elements.

These findings demonstrate that GATA1 directly activates HLTF transcription, HLTF forms a transcription complex with GATA1, and HLTF loss impairs GATA1’s genomic binding. These reciprocal interactions form a positive feedback loop that reinforces the transcriptional program driving erythroid development.

### HLTF is dysregulated in erythroid disorders and promotes erythropoiesis in patient-derived cells

**Figure 6.**
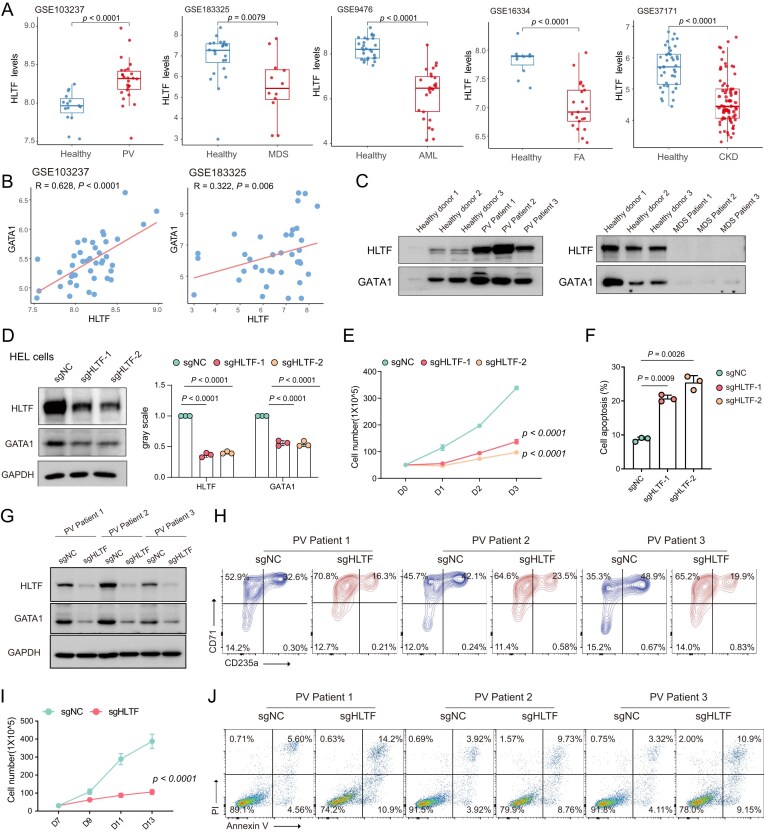
HLTF expression and functional impact in erythroid disorders and PV patient-derived cells. (**A**) Analysis of public transcriptomic datasets of HLTF expression levels in various hematological diseases compared with healthy controls. (**B**) Correlation analysis between HLTF and GATA1 expression in PV (GSE103237) and MDS (GSE183325) datasets. (**C**) Western blot analysis of HLTF and GATA proteins in bone marrow mononuclear cells (BMMCs) from clinical PV and MDS samples. (**D**) HLTF and GATA1 protein expression in HLTF KO HEL cells. (**E**) Cell proliferation curves of HEL cells following HLTF knockout. (**F**) Cell apoptosis analysis based on Annexin V/PI staining. (**G**) HLTF and GATA1 protein expression in erythroid cells from PV patients following HLTF knockout. (**H**) Flow cytometric analysis of CD71 and CD235a expression in PV-derived erythroid cells after HLTF knockout. (**I**) Cell proliferation in HLTF-deficient PV-derived erythroid cells. (**J**) Apoptosis assessment in HLTF KO PV-derived erythroid cells.

To assess the clinical relevance of HLTF in erythroid-related diseases, public transcriptomic datasets were analyzed. HLTF expression was found to be significantly elevated in PV and down-regulated in multiple anemia-related disorders, including MDS, acute myeloid leukemia (AML), Fanconi anemia (FA), and chronic kidney disease (CKD) (Fig. 6A). Correlation analysis demonstrated a strong positive relationship between HLTF and GATA1 expression in both PV (GSE103237) and MDS (GSE183325) cohorts (Fig. 6B). Western blot analysis of primary bone marrow patient samples confirmed that HLTF and GATA1 were up-regulated in PV patients and down-regulated in MDS patients compared with healthy donors (Fig. 6C).

Expression profiling using the RDDC database (https://rddc.tsinghua-gd.org/) showed that HLTF was most highly expressed in the erythroid leukemia cell line HEL (Supplementary Fig. S8). HLTF knockout in HEL cells led to a marked reduction in HLTF and GATA1 protein levels (Fig. 6D). Cell growth curve analysis and flow cytometry demonstrated that HLTF loss suppressed proliferation and increased apoptosis in HEL cells (Fig. 6E, F).

To further investigate the functional role of HLTF in PV, HLTF was knocked out in CD34^+^-derived erythroid cells from three PV patients. Western blot confirmed reduced HLTF and GATA1 expression in HLTF-deficient cells (Fig. 6G). Flow cytometry revealed a decreased proportion of CD71^+^/CD235a^+^ erythroid cells (Fig. 6H), while cell proliferation was significantly impaired during erythroid differentiation (Fig. 6I). Annexin V/PI staining indicated increased apoptosis following HLTF knockout (Fig. 6J).

### HLTF maintains chromatin accessibility and GATA1 function in erythroid cells from PV patients

**Figure 7.**
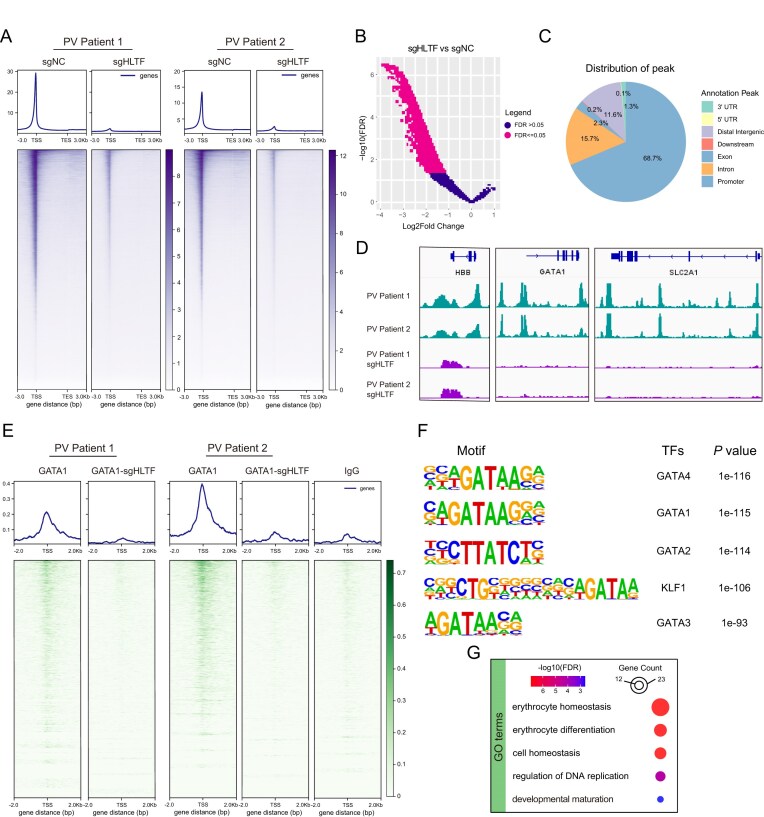
HLTF maintains chromatin accessibility and GATA1 function in erythroid cells from PV patients. (**A**) Aggregated ATAC-seq signal plot in HLTF-deficient and control erythroid cells from PV patients. (**B**) Differentially expressed peaks between sgNC groups and sgHLTF groups. (**C**) Pie chart illustrating the genomic distribution of down-regulated peaks. (**D**) IGV browser tracks showing chromatin accessibility at representative erythroid gene loci (HBB, GATA1, and SLC2A1) in HLTF-deficient and control cells. (**E**) Aggregated CUT&Tag signal plot of GATA1 binding around TSSs in HLTF-deficient and control PV-derived erythroid cells. (**F**) Motif enrichment analysis of genomic regions with reduced GATA1 occupancy after HLTF depletion. (**G**) GO enrichment analysis of genes associated with regions showing decreased GATA1 binding.

To determine whether the molecular mechanisms of HLTF in patient-derived erythroid cells resemble those observed under physiological conditions, ATAC-seq was performed in CD34^+^-derived erythroid cells from PV patients following HLTF knockout. Aggregated signal plots and heatmaps revealed a global reduction in chromatin accessibility, particularly around TSSs (Fig. 7A). Differential peak analysis identified 3524 regions with significantly decreased accessibility (Fig. 7B). Genomic annotation showed that 68.7% of these down-regulated peaks were located in promoter regions (Fig. 7C). IGV browser views demonstrated reduced promoter accessibility at representative erythroid genes, including HBB, GATA1, and SLC2A1 (Fig. 7D). Motif analysis of down-regulated peaks revealed a significant loss of GATA motif accessibility (Supplementary Fig. S9A).

Subsequently, we conducted CUT&Tag analysis for GATA1 in these HLTF-deficient CD34^+^ erythroid cells. Aggregated signal plots and heatmaps demonstrated a global reduction in GATA1 chromatin occupancy around TSSs following HLTF knockout (Fig. 7E). PCA results further highlighted a global shift in GATA1 peak occupancy after HLTF knockout (Supplementary Fig. S9B). Genomic structural annotation indicated a broad distribution of these differential peaks across promoters, distal intergenic regions, and introns (Supplementary Fig. S9C). Enrichment analysis of down-regulated peaks revealed a significant enrichment of GATA motifs (Fig. 7F). Furthermore, gene annotation followed by enrichment analysis indicated that regions with reduced GATA1 binding were significantly associated with pathways involved in erythrocyte differentiation, erythrocyte homeostasis, and DNA replication (Fig. 7G).

## Discussion

In this study, HLTF was identified as a critical transcriptional regulator of erythropoiesis, exerting its effects through both direct activation of GATA1 and cooperative engagement with the erythroid transcriptional program. Loss-of-function studies demonstrated that HLTF deficiency led to significant down-regulation of GATA1 at both the mRNA and protein levels, accompanied by impaired proliferation, increased apoptosis, and delayed erythroid differentiation. These phenotypes were further validated *in vivo* using BMT models, where HLTF knockdown resulted in anemia, reduced erythroid output, and compensatory splenic hematopoiesis. These findings are consistent with single-cell RNA-seq data, which showed that HLTF expression is enriched in late-stage erythroid cells, reinforcing its lineage-specific role.

To date, most studies of GATA1 regulation have focused on post-transcriptional mechanisms, including regulation by heat shock proteins, ribosomal proteins, and deubiquitinases [8, 38–40]. In this study, we address a critical gap by investigating the upstream transcriptional regulation of GATA1 and identifying HLTF as a previously unrecognized TF and chromatin regulator in this context. During erythroid development—particularly terminal differentiation—erythrocytes undergo substantial chromatin structural reorganization [41–44]. Through multi-omics analyses, we demonstrate that HLTF plays a pivotal role in shaping the erythroid transcriptional program and chromatin landscape in both healthy and PV-derived erythroid cells. These findings highlight HLTF as a transcriptional and epigenetic regulator uniquely positioned to control lineage-specific gene expression during erythroid maturation. Notably, DNA pull-down MS identified numerous additional proteins at the GATA1 promoter. Future studies will be needed to determine whether any of these factors, beyond HLTF, directly bind and functionally contribute to GATA1 regulation.

Notably, the incomplete rescue of HLTF-deficient phenotypes by GATA1 overexpression suggests that HLTF may also contribute to erythropoiesis through GATA1-independent mechanisms. Previous studies have demonstrated that GATA1 and TAL1 interact with BRG1 to regulate erythroid gene expression [45–47]. Our findings extend this model by identifying HLTF as a non-canonical SWI/SNF-related ATPase that associates with the GATA1–TAL1 complex and promotes GATA1 occupancy at erythroid promoters.

Interestingly, recent studies have shown that HLTF can inhibit the accumulation of G4s [20]. G4 structures are known to impede chromatin accessibility by blocking RNA polymerase progression and inducing hypomethylation of CpG islands [48–50]. Moreover, G4 accumulation promotes DNA damage and genomic instability [51–53]. Therefore, HLTF’s DNA binding activity may depend on local DNA topology, and its recruitment to the GATA1 promoter might be influenced by higher-order chromatin structure and interactions between proximal and distal regulatory elements.

An important finding of this study is the identification of a reciprocal regulatory loop between HLTF and GATA1. Mechanistically, CUT&Tag profiling revealed that HLTF knockout significantly impaired the ability of GATA1 to bind to its target sites across the genome, indicating that HLTF is required for proper chromatin engagement by GATA1. Whether this regulatory dependence is bidirectional remains to be determined. A logical next step would be to perform CUT&Tag profiling of HLTF following GATA1 depletion, to assess whether GATA1 also facilitates HLTF genomic localization or stability at erythroid regulatory elements. Furthermore, Co-IP experiments revealed that HLTF and GATA1 associate through defined domains—specifically, the HIRAN, DEXD, and RING domains of HLTF and the NF-CF region of GATA1. While this interaction underscores their cooperative function, further mapping at the amino acid level is needed to pinpoint the core residues essential for binding. In addition, whether HLTF interacts with GATA1’s interacting partners, such as HES6, deserves further study.

The dysregulation of HLTF in erythroid-related diseases suggests that it may serve as both a mechanistic driver and a potential biomarker. In PV, elevated HLTF expression probably contributes to excessive erythropoiesis by enhancing GATA1-dependent transcription and chromatin accessibility at erythroid loci. Our finding that HLTF knockout in PV-derived erythroid cells suppresses erythroid hyperplasia underscores its potential role in disease pathogenesis. Conversely, in disorders such as MDS, down-regulation of HLTF may exacerbate ineffective erythropoiesis by weakening GATA1-mediated transcriptional control. This dual role—promoting erythroid expansion when overexpressed and impairing maturation when deficient—positions HLTF as a critical rheostat of erythroid lineage output. Clinically, HLTF expression profiling may aid in disease classification or risk stratification, distinguishing hyperproliferative states from hypoplastic or dysplastic conditions. Furthermore, targeted modulation of HLTF activity—either through small molecule inhibitors in PV or through epigenetic reactivation in MDS—could represent a novel therapeutic avenue. Given HLTF’s upstream regulatory influence on GATA1 and its cooperative role in chromatin remodeling, future efforts to dissect HLTF–GATA1 interaction domains may enable the development of precision therapeutics aimed at restoring balanced erythropoiesis in diverse clinical settings.

In conclusion, our findings establish HLTF as a novel upstream regulator of GATA1, acting through both transcriptional activation and protein–protein interaction mechanisms. HLTF enriches the regulatory network centered on GATA1 and plays an essential role in erythroid development. These findings advance our understanding of RBC biology and open up new avenues for the diagnosis and treatment of erythroid disorders.

## Supplementary Material

gkaf1506_Supplemental_File

## Data Availability

The identified succinylated protein data have been deposited in the National Genomics Data Center (https://ngdc.cncb.ac.cn/) with the dataset identifier PRJCA043455. Further inquiries may be directed to the corresponding author.
