# Supplementary material for: HLTF cooperates with GATA1 to activate transcriptional programs and chromatin remodeling during erythroid development
Source: Nucleic Acids Res. 2026 Jan 9;54(1):gkaf1506. doi: 10.1093/nar/gkaf1506 (PMC12784948; doi:10.1093/nar/gkaf1506)
Supplement: gkaf1506_Supplemental_File [file gkaf1506_supplemental_file.pdf]

Supplemental Methods

Cell culture and erythroid differentiation

Human CD34+ hematopoietic stem and progenitor cells (HSPCs) were isolated from umbilical cord blood or peripheral blood samples using magnetic-activated cell sorting (MACS) or other standard protocols. CD34+ cells were cultured at 1 × 105 cells/ml in Iscove's Modified Dulbecco's Medium (IMDM; Life Technologies) supplemented with 200 μg/ml human holo-transferrin (Sigma-Aldrich), 2% human AB plasma, 10 μg/ml insulin (Sigma-Aldrich), 3% fetal bovine serum (FBS; Gibco, Thermo Fisher), 3 IU/ml heparin (Qilu Pharma), and 1% penicillin/streptomycin (Thermo Fisher). During Days 0–6, the medium additionally contained 1 ng/ml interleukin-3 (IL-3), 10 ng/ml stem cell factor (SCF; STEMCELL Technologies), and 3 IU/ml erythropoietin (EPO). From Days 7–11, cytokine supplementation consisted solely of 1 IU/ml EPO and 10 ng/ml SCF. On Day 11, the cell concentration was adjusted to 1 × 106 cells/ml, and further adjusted to 5 × 106 cells/ml on Day 14.

For human umbilical cord-derived progenitor erythroid 2 (HUDEP-2) cells, Thaw and plate HUDEP2 cells in expansion medium at a density of 100,000 cells/mL. Culture Medium: StemSpan™ SFEM (Serum-Free Expansion Medium; STEMCELL Technologies) supplemented with 50 ng/mL recombinant human stem cell factor (SCF), 3 IU/mL erythropoietin (EPO), 0.4 μg/mL dexamethasone, 1 μg/mL doxycycline, and 2% Penicillin-Streptomycin solution (10,000 U/mL stock). Cells typically double every 24–36 hours. Change medium every 3–4 days, maintaining cell density below 800,000 cells/mL. For erythroid differentiation, HUDEP2 cells were cultured in Iscove's Modified Dulbecco's Medium (IMDM) supplemented with 330 μg/mL human holo-transferrin, 10 μg/mL recombinant human

insulin, 2 IU/mL heparin, 5% inactivated human plasma, 3 IU/mL erythropoietin (EPO), 2% Penicillin-Streptomycin solution (10,000 U/mL stock), and 1% L-glutamine (added supplementally to the basal L-glutamine content of IMDM).

CRISPR-Cas9 gene editing

Single-guide RNAs (sgRNAs) targeting the HLTF gene were designed using the CRISPR design tool (http://www.crispr.mit.edu/) and synthesized. The sgRNAs were cloned into the lentiCRISPR v2 vector (Addgene, Cambridge, MA, USA), which co-expresses the Cas9 nuclease and a puromycin resistance gene for selection.

Human HSPCs and HUDEP-2 cells were transduced with lentiviral particles containing the sgRNA-Cas9 expression construct using standard viral transduction protocols. Briefly, cells were plated at a density of 1 x 10^6 cells/mL in the presence of polybrene (4 μg/mL), followed by incubation with lentiviral supernatant. After 24 hours, cells were subjected to puromycin selection (1 μg/mL) for 3 days to enrich for successfully transduced cells.

Luciferase reporter assay

To assess the functional activity of HLTF on the GATA1 promoter, a luciferase reporter assay was performed. The wild-type and mutant versions of the GATA1 promoter region were cloned into the pGL3-Basic luciferase reporter vector (Promega, USA). The mutant version of the GATA1 promoter contained mutations in the HLTF binding motif, which were introduced using site-directed mutagenesis (Agilent Technologies, USA). For the assay, HEK-293T cells (1 x 10^6 cells/well) were seeded in 24-well plates and co-transfected with 1 μg of the pGL3-GATA1

luciferase reporter plasmid (wild-type or mutant) and 100 ng of a Renilla luciferase reporter plasmid for normalization of transfection efficiency. Transfections were carried out using Lipofectamine 3000 reagent (Thermo Fisher Scientific) according to the manufacturer's instructions. After 24 hours, cells were treated with or without overexpression constructs for HLTF and/or GATA1, as described in the manuscript.

Following transfection and treatment, luciferase activity was measured using the Dual-Luciferase Reporter Assay System (Abbkine). The firefly luciferase activity (which reflects the GATA1 promoter activity) and Renilla luciferase activity were measured using a GloMax Luminometer (Abbkine). Firefly luciferase activity was normalized to Renilla luciferase activity to correct for variations in transfection efficiency. For the analysis, the relative luciferase activity was calculated as the ratio of firefly to Renilla luciferase activities, and data were presented as fold-change compared to the control group. Mutations in the HLTF binding motif abolished HLTF binding and its ability to activate the GATA1 promoter, as evidenced by the loss of luciferase activity in the mutant GATA1 promoter construct.

Flow cytometry

Flow cytometry was used to assess cell proliferation, apoptosis, cell cycle distribution, and erythroid differentiation at various stages of differentiation in both HLTF knockout and control cells.

Proliferation and apoptosis analysis:

For apoptosis analysis, cells were stained with Annexin V and propidium iodide (PI) following the manufacturer's instructions (Annexin V-FITC Apoptosis Detection Kit, Vazyme)). Annexin

V binding was used to detect early apoptotic cells, while PI staining was used to identify late apoptotic or necrotic cells. The samples were analyzed using a BD FACSCanto II flow cytometer (BD Biosciences), and data were analyzed using FlowJo software.

Cell cycle analysis:

To assess cell cycle distribution, cells were stained with PI (10 μg/mL) and analyzed for DNA content. After fixation in 70% ethanol overnight, cells were treated with RNase A (100 μg/mL) for 30 minutes at 37°C, followed by incubation with PI (10 μg/mL) for 30 minutes at room temperature. Flow cytometric analysis was performed to measure the DNA content in individual cells, allowing the identification of cells in the G0/G1, S, and G2/M phases of the cell cycle. The data were analyzed using FlowJo software to determine the percentage of cells in each phase.

Flow cytometry sorting

Specifically, after HLTF or GATA1 knockdown, $CD71^{+}CD235a^{+}$ erythroblast populations were sorted and performed bulk RNA-seq.

Peripheral Blood Analysis

Peripheral blood (PB) was collected from recipient mice at 4 weeks post-transplantation for complete blood count (CBC) analysis, including RBC count, hemoglobin (Hb) levels, and hematocrit (HCT). These parameters were measured using an automated hematology analyzer (Sysmex).

Bone Marrow and Spleen Analysis

At the end of the transplantation period, mice were euthanized, and their femurs, tibias, and spleens were harvested. Bone marrow was flushed from the long bones and processed for flow cytometry analysis to assess erythroid and myeloid differentiation. The spleens were also analyzed to assess extramedullary hematopoiesis, as indicated by changes in spleen size and weight. Flow cytometry was performed to analyze the percentage of mature RBCs and other erythroid stages (e.g., polychromatic and orthochromatic erythroblasts) in both the bone marrow and spleen using antibodies against, CD44, CD45, and TER-119, which are markers of erythroid differentiation.

HSPC Isolation and Transduction

C-kit^{+} HSPCs were isolated from the femurs and tibias of 8–10 week old WT mice using the MACS® Cell Separation Kit (Miltenyi Biotec, Auburn, CA, USA). The isolated HSPCs were transduced with lentiviral vectors expressing short hairpin RNAs (shRNAs) targeting Hltf or a non-targeting control (shNC) in a spinoculation process, following the manufacturer's protocol. After transduction, cells were expanded in culture medium containing StemSpan™ SFEM (STEMCELL Technologies) supplemented with growth factors, including SCF (50 ng/mL), TPO (50 ng/mL), IL-3 (25 ng/mL), IL-6 (25 ng/mL), and FLT3L (50 ng/mL) for 48 hours.

Transplantation Procedure

Transduced HSPCs (1 x 10^6 cells) were transplanted into lethally irradiated (9 Gy) recipient mice. Mice were allowed to recover for 4–6 hours post-irradiation and then injected intravenously with the transduced HSPCs. Following transplantation, the mice were monitored

for recovery and allowed to engraft for 4 weeks before analysis.

Bone marrow mononuclear cell (BMMC) collection and CD34$^+$ cell isolation

Bone marrow aspirates were diluted 1:1 with PBS containing 2% fetal bovine serum (FBS) and layered over Ficoll (sigma) in 15 mL tubes. Samples were centrifuged at 300 × g for 30 minutes at room temperature without brake. The mononuclear cell layer was collected from the interface, washed twice with PBS + 2% FBS, and centrifuged at 300 × g for 10 minutes.

The isolation of patients-derived CD34$^+$ HSPC was performed referred to the protocol in healthy donors.

Differential analysis

Differential gene expression analysis was performed using the DESeq2 package in R. Genes with a false discovery rate (FDR) adjusted p-value < 0.05 and fold change ≥ 0.25 were considered differentially expressed. Gene set enrichment analysis (GSEA) was conducted to identify biological pathways and processes associated with HLTF depletion. Gene ontology (GO) enrichment analysis was also performed to categorize the differentially expressed genes involved in erythropoiesis, cell cycle regulation, and other relevant cellular processes.

Co-immunoprecipitation (Co-IP)

Co-IP assays were performed to investigate the physical interaction between HLTF and GATA1 in erythroid cells. Day 9 (D9) erythroid cells were collected and lysed using RIPA buffer (50 mM Tris-HCl, pH 7.4, 150 mM NaCl, 1% NP-40, 0.5% sodium deoxycholate, 1 mM EDTA,

and 1x protease inhibitor cocktail; Thermo Fisher Scientific). The lysates were incubated for 30 minutes on ice and then cleared by centrifugation at 12,000 × g for 10 minutes at 4°C to remove cell debris. Protein concentrations were measured using the BCA Protein Assay Kit (Thermo Fisher Scientific).

To identify HLTF-GATA1 interactions, protein lysates (500 μg) were incubated overnight at 4°C with primary antibodies against HLTF (Bethyl) or GATA1 (Cell Signaling Technology), followed by the addition of protein-A/G agarose beads (Selleck) for 8 hours at 4°C. The antibody-protein complexes were washed extensively with RIPA buffer to remove non-specifically bound proteins.

After washing, the immunocomplexes were eluted by incubating the beads in PBSTbuffer for 30 minutes at 37°C. The eluates were analyzed by SDS-PAGE and Western blotting using specific antibodies against HLTF, GATA1, and other relevant proteins to confirm the presence of the interacting complex.

To verify the specificity of the interaction, control immunoprecipitations were performed using non-specific IgG antibodies. The intensity of the bands was quantified using ImageJ software, and the relative interaction between HLTF and GATA1 was determined by comparing the levels of HLTF or GATA1 in the immunoprecipitated complex with input samples.

Electrophoretic mobility shift assay (EMSA)

DNA–protein binding was assayed with the LightShift™ Chemiluminescent EMSA Kit (Thermo Fisher Scientific, No. 20148) following the manufacturer's protocol, with minor adjustments. The double-stranded probe corresponded to the GATA1 binding site:

WT: 5′-Biotin-TTGACCTGCTCTCAGTGAGATAATCTGCTGAGCCTGAGTCTGCC-3′;
Cold probe: 5′-GGCAGACTCAGGCTCAGCAGATTATCTCACTGAGAGCAGGTCAA-3′.
Complementary strands were mixed at equimolar concentrations in 1× annealing buffer, heated to 95 °C for 5 min, and slow-cooled to room temperature to generate the dsDNA probe. For competition assays, an unlabeled ("cold") duplex of the same sequence was prepared identically. Binding reactions (20 μL) contained 1× EMSA binding buffer (10 mM Tris-HCl pH 7.5, 50 mM KCl, 1 mM DTT), 2.5% glycerol, 0.05% NP-40, 50 ng/μL poly(dI·dC), 20 fmol biotin-labeled probe, and purified recombinant proteins as indicated (HLTF, GATA1, or both). For specific competition, a 25× molar excess of unlabeled duplex was added and pre-incubated with proteins for 10 min before adding the biotinylated probe. Reactions were incubated 20 min at room temperature and resolved on 6% native polyacrylamide gels in 0.5× TBE at 100 V. DNA–protein complexes were transferred to a positively charged nylon membrane, UV-crosslinked, and detected by chemiluminescence using streptavidin–HRP and the kit substrate.

Statistical analysis

All statistical analyses were performed using GraphPad Prism software (version 8.0, GraphPad Software, San Diego, CA, USA). Data are presented as means ± standard deviation (SD) or means ± standard error of the mean (SEM), as indicated in the figure legends. The normality of the data was assessed using the Shapiro-Wilk test, and all data were found to be normally distributed. For comparisons between two groups, statistical significance was determined using a two-tailed unpaired t-test. For multiple group comparisons, one-way or two-way analysis of variance (ANOVA) was performed, followed by post hoc analysis using Tukey's or Sidak's

multiple comparison tests, as appropriate. For all analyses, a p-value of <0.05 was considered statistically significant. Significance levels are indicated as follows: *p < 0.05, **p < 0.01, ***p < 0.001, and ****p < 0.0001.

Supplemental Tables and Figures

Supplemental Table 1. All antibodies used in this study

Antibodies	Company	Catolog
Rabbit anti-SMARCA3 Antibody, Affinity Purified	Bethyl	A300-230A-T
GATA-1 (D52H6) XP ® Rabbit mAb	Cell Signaling Technology	#3535
GAPDH Antibody	Santa Cruz Biotechnology	sc-137179
Annexin V-FITC/PI Apoptosis Detection Kit	Vazyme	A211-01
Cell Cycle Staining Kit	MULTI SCIENCES	CCS012
BD Horizon™ BV421 Mouse anti-Human CD235a antibody	BD Biosciences	No. 562938
BD Pharmingen™ PE Mouse Anti-Human CD71 antibody	BD Biosciences	561938
BD Pharmingen™ PE Rat Anti-Mouse CD71	BD Biosciences	567206
BD Pharmingen™ APC-Cy™ 7 Rat Anti-Mouse CD45	BD Biosciences	561037
BD Pharmingen™ FITC Rat Anti-Mouse TER-119/Erythroid Cells	BD Biosciences	561032
BD Pharmingen™ APC Rat Anti-Mouse CD44 antibody	BD Biosciences	561862
TER-119 Antibody	Thermo Fisher	14-5921-82

F4/80 Rabbit mAb	Abclonal	A23788
CD11b/ITGAM Rabbit mAb	Abclonal	A23508
High-Sensitivity Open Chromatin Profile Kit 2.0 (for Illumina®)	novoprotein	N248
NovoNGS® CUT&Tag 4.0 High-Sensitivity Kit (for Illumina®)	novoprotein	N259-YH01
Histone H3K27ac antibody (pAb)	proteintech	39135
Normal Rabbit IgG	Cell Signaling Technology	2729
Rabbit anti GFP-Tag mAb	Abclonal	AE078
Anti-DDDDK-tag pAb	MBL	PM02
HSC70 antibody (W27)	Santa Cruz Biotechnology	sc-24
Ficoll-Paque PLUS	GE Healthcare	17-1440-03
2×Universal SYBR qPCR Master Mix	YoungGen	QP101A
High Performance qRT Master Mix(+gDNA Remove)	YoungGen	RT201
Dual Luciferase Reporter Gene Assay Kit	Abbkine	KTA8010

Supplemental Table 2. The sequences of primers, shRNA, and sgRNA

Names	Sequences (5´- 3´)
**HLTF**	
Forward	AGATGGACTAAGCAAAGACGCA

Reverse	TTTCTTCTTTTGGGGCGGGA
**GATA1**	
Forward	CTGTCCCCAATAGTGCTTATGG
Reverse	GAATAGGCTGCTGAATTGAGGG
**Hltf**	
Forward	TCCTATACGTTCACGAGGGGT
Reverse	GCTCTCGTTGTAAAGCAACC
**Gata1**	
Forward	GGCAAGACGGCACTCTACC
Reverse	CAAGAACGTGTTGTTGCTCTTC
**TFRC**	
Forward	ATCGGTTGGTGCCACTGAATGG
Reverse	ACAACAGTGGGCTGGCAGAAAC
**SLC4A1**	
Forward	CTGCTGGTGTTTGAGGAAGCCT
Reverse	CACCAGCAGGATGAGCCAGAAG
**SLC2A1**	
Forward	TTGCAGGCTTCTCCAACTGGAC
Reverse	CAGAACCAGGAGCACAGTGAAG
**ALAS2**	
Forward	GCCTCAAAGGATGTGTCCGTCT
Reverse	TACTGGTGCCTGAGATGTTGCG
**EPB42**	
Forward	ACCCAAGTGCTCCTAATGGAGG
Reverse	CCATCCTCACAGCACTTCCAGA
**HBB**	
Forward	CACCTTTGCCACACTGAGTGAG
Reverse	CCACTTTCTGATAGGCAGCCTG
**sgHLTF-1**	ACATCTTGGAATTCAAAACG
**sgHLTF-2**	ACGAGTAAACACAAATCCAG

**shHltf-1**	CAAGTATATACAGACTATAA
**shHltf-2**	CAGCGCAATGACTTATATTAT

Supplemental Table 3 GATA1 promoter sequences

GATA1 >NC_000023.11:48784590-48786589 HS chromosome X, GRCh38.p14

AATAGGGGTACTTAATATTTATTGAGATTTCAGATTCAAGTAAGTTAAGATCATGTAACA
TGGGTTAAAACTCATTTAGTCTCCCCAGTCACTGCATGGAGTGGCTTATTGGGGTATATT
ATTACCCCATTTTTACAGATGAGGAGCTAAATTCCAGAGGGGATGGTAACTCTCCCAAG
GTCACCTGATCCATTAGTGGTTAGGGCAGTCTATGAACGAGGATGAGCTGGCTCTAGA
GACCATCTCCTGAAACCACTGCCACTTAAGTCAGAGGTGTCTTTGTATTCGTTCTCCTT
ATTACTGATGAGGGTTCAGCATGGGGCGTGGCACACCAGCAGGAGCTCAATAAATAGG
AATTCATGGGCTTGCTCTCTTTCCTCCTCAAATCTCCCATTGCATATGAGGACACTGAGG
CTCAGAGGAGTGAGATAAGAATGGTAAGATCCCTCCAGGGTGGGCCAGTGGGCAAGC
TGACCCCACTGACCTGGGGCTCCACCCTTGGCCTCTGCCCTTTGTAGGTCTCAGTCTCC
CCATCTGTAGACTAGGTGGGCAGGACAGCTCCTTGTCCCACGTGTGCATGTGTGTCAG
ATGCTTTCATATAGAAAAAGTGCTCAGGTTAGGTACAAGTCTATGTGAGGTCTATGAGA
CACTGTGGTTGTTGTCATTAATATTTGGAGGAGGTGGGGCAGGGCAAGAGTGTGGGGA
CTGGGGTGCGGAGGCCTAGCCACATTCTGGTTGTCCCCTGCTGAGGGCTGAGGGCAGA
GCCACAGGCTACATCAATCCACATCCTCCCATCCTACCTGCATGGGCACCACATGCCCA
GGGTGTGATCCAGTCTGCCTGGACCTTCTTCTGAGTCTGTCTCTGAATATCCCTCTGTCT
CTGTCTCCCTTCTCAGTTCTCCACTTCTGTCTTTGGCCTCTTTCCCTTTCAGGTTTTATCT
GTCACTTCTGTCCATCTCTGTCTCTCATTCTCTCTTTGCCTCTCTCTATATGTCTTTAATG
GTCTCTGGTTCTCCCCGGGTGTCTCGCCCTCTTTGTCTCTCCTTTTCTTTTGTGCCCTCT

CTCTGCCTCTTTCCACGTCTGTCTCCTTCTCTATTCATCTCTGTCACTCTCTTTCTCCCCA
TTTCCATCTCTCTCCGCCTATCTCTGTTTTTGTTTGTCTCTCTGCCTCTGTCTCTCCCACT
CCACCCCTTTCCTTTCCTACCCTATACCACTCCTCGAGGAATCATCCCTGGCTCCCACCT
CAGTTTCCCGCCTCCAAGGCAGCATGGCGGGCAAGAAGTTGAGGCCACTGTCCCTGG
GTGTTCCTACCCCCACACCCTCACCCCAAGACAGCCTGTTACTGCGGCGCCAACAGCC
ACGGTCGCCTACATCTGATAAGACTTATCTGCTGCCCCAGGGCAGGCCGGAGCTGGCG
TAAGCCCCAGTGGGGCGCTAAGTGAGTGTGCCCCTGCCTCCCGCCAGCACTGGCCTGG
CCTGCAGGCTTAGCCTGGGTCATCAAGGTATCCCACAGGCTCTAGTTCAAATCCAGCA
GAACCTCTCTGAGCCTCACTCTTCTCACCTGCAAAATGGGTACAGCCACATCCCTTCTC
TCCCTGCAGCCAGGAAGACGCACATACACAGGAGTCTAGCCCACACCGGCCCCGCAC
AAATTAAGGGCTTTACTCTCTGAAAAGCCCAGTGAAGTCATGAAACCATATCTGCTATT
TTCATTTATCTTGGTTTCAGCCTATTTTGCTTGTCTGGACACTACAGTCCACGGGAGCCT
AGGTCGAGCGAGGTCCAAGAATCCCCAGGGTGGGCAGGGAGGGTGGAAGAGGGCCT
CCAGTGCCCAAGAGGTGCCCCACAAGCATGGGACCCGCCCCCTCCCCTGGACTGCCC
CACCCACTGGGGCACCAGCCACTCCCTGGGGAGGAGGGAGGAGGGAGAAGGGAGGG
AGGGAGGGAGGGAGGAAGGGAGCCTCAAAGGCCAAGGCCAGCCAGGACACCCCCTG
GGATC

Supplemental Table 4. The list of proteins identified at the GATA1 promoter (DNA pull-down MS results, Top 100)

Gene names	Unique peptides	Unique peptides	Sequence coverage Scramble [%]	Sequence coverage GATA1 promoter [%]

	Scramble	GATA1 promoter		
DDB1	0	24	0	23.3
HLTF	0	23	0	27.2
WRN	0	15	0	12.6
HNRNPAB	0	14	0	25.9
HNRNPR	0	12	0	26.2
RECQL	0	12	0	22.2
WDR76	0	12	0	18.7
SSBP1	0	10	0	59.5
TOP3A	0	10	0	10.5
SMARCAL1	0	10	0	10.9
SYNCRIP	0	9	0	21.7
YBX1	0	9	0	51.2
LIG3	0	8	0	7.7
DDB2	0	8	0	23.7
PURB	0	8	0	26.9
MYL12A	0	7	0	40.9
NUP155	0	7	0	6.6
TFAM	0	7	0	29.7
HNRNPA0	0	7	0	24.9
DNAJC9	0	7	0	27.3

MYO1G	0	6	0	8.8
PFKL	0	6	1.2	12.7
MPG	0	6	0	23.5
CIRBP	0	6	0	45.3
LYPLA2	0	5	0	32.5
RPN2	0	5	0	13.2
SERPINB2	0	5	0	12.5
PURA	0	5	0	23.6
RBM39	0	5	0	11.5
DDX1	0	5	0	8.5
UHRF1	0	5	0	6.3
TREX1	0	5	0	12.7
APOBEC3B	0	5	0	15.7
RTCB	0	5	0	14.5
SQRDL	0	5	0	15.1
HNRNPDL	0	4	0	11
PABPC1	0	4	0	7.7
RPA3	0	4	0	47.1
MSH2	0	4	0	4.8
QARS	0	4	0	5.7
RPS11	0	4	0	21.5
RAE1	0	4	0	15.8

RBM3	0	4	0	27.4
FKBP8	0	4	0	13.3
SF3B3	0	4	0	3.9
ACO2	0	4	0	6.9
APOBEC3C	0	4	6.3	30
SLC25A13	0	4	0	6.2
ARPC2	0	3	0	9.7
RNASEH1	0	3	0	14.3
PMPCB	0	3	1.4	9.4
FLOT1	0	3	0	7.5
NDUFA10	0	3	0	9.9
GLUD1	0	3	0	5.7
ASNS	0	3	0	5.2
CAD	0	3	0	1.6
RFC1	0	3	0	3
DLST	0	3	0	8.8
CPT1A	0	3	0	4.4
DNM2	0	3	0	2.9
RPS7	0	3	0	16
SRSF7	0	3	0	17.6
SERPINB12	0	3	0	8.1
VAT1	0	3	0	13

RBM42	0	3	0	7.5
RMI1	0	3	0	5.9
TECR	0	3	0	9.7
USP24	0	3	0	1.5
CLIC1	0	2	0	11.2
PSMD3	0	2	0	5.1
H2AFY	0	2	0	9.9
SNRNP200	0	2	0	1
FADS2	0	2	0	4.1
HSPB1	0	2	0	12.7
HIST1H2AC	0	2	17.7	35.4
HIST1H2BB	0	2	12.7	44.4
H2AFV	0	2	12.5	31.2
PEPD	0	2	0	5.3
FAH	0	2	0	5.3
YBX3	0	2	0	33.1
RCC1	0	2	0	3.8
SRM	0	2	0	7
MSH3	0	2	0	1.8
DDX6	0	2	0	6.4
VARS	0	2	0	1.6
FUS	0	2	2.7	10.5

ATP6V1A	0	2	0	3.6
EIF4A3	0	2	5.6	11.9
HELZ	0	2	0	1.3
GCLC	0	2	0	3.1
BLM	0	2	0	1.4
RARS	0	2	0	3.5
HADHB	0	2	0	3.8
SPCS3	0	2	0	11.7
RAB10	0	2	5.5	17
ARF3	0	2	6.1	34.8
ACTA1	0	2	25.2	35.3
EWSR1	0	2	0	4.4
SP3	0	2	0	2.2
ILF2	0	2	0	6.4

Supplemental Table 5. Overlap of HLTF targets and GATA1 targets

SLC4A1	ANK1	SLC25A37	ADD2	KCNH2	SPTB	ERFE	DDIT4
PIM1	OSBP2	SLC1A5	ALAS2	ABCB10	TFDP1	HEMGN	HBD
MYBL2	TUBA1B	RHAG	DMTN	IQSEC1	SLC7A5	HK1	ABCC5
UBAC1	TAL1	XK	FADS2	MYH10	PRDX2	TLCD4	MCM2
FECH	ALAD	STEAP3	APOL4	CPOX	LBH	SLC2A1	FHDC1

TSPAN17	CDC20	MFHAS1	NCS1	HBB	ANKLE1	ACSS1	KIF18B
NUDT4	MYC	TK1	CCNF	SLC6A8	HNRNPAB	TRAK2	KIFC1
RBM38	TUBG1	TTLL12	NCEH1	HSPA6	E2F2	FEN1	GUCD1
TUBB2A	SYNGR1	BIRC5	TMEM14C	GYPA	ARHGAP23	SLC29A1	TANGO2
KLHL18	GAS2L1	SLC38A5	PYCR1	OAT	UROS	ESPL1	ERMAP
LIG1	SLC41A1	MFSD2B	A4GALT	RHD	DYRK3	GYPB	CDC42EP4
BLVRA	DNAJC9	CDCA4	FADS1	HEBP1	ELOVL6	SPC24	FAM210B
AGFG2	METTL13	SH3TC2	CA2	QSOX2	REXO2	HES6	KAT2B
CHST2	PFKM	TOMM40	SLC6A9	VEGFA	PFAS	TNKS1BP1	GYPE
CDC25A	CCNE1	TRIM10	CDK4	RPS6KL1	ARHGAP19	PRKAR2B	DNAJC6
RAB3IL1	KANK2	RCL1	SEC14L4	STAB1	POMGNT2	ACAT1	CDKN2C
CDCA3	UBE2T	PHOSPHO1	DEPDC1B	SLC22A23	RASGRP3	AIFM2	ZDHHC14

SDC4	E2F1	KREMEN1	PCK2	KIF26A	DTYMK	FN3K	TGM2
FHL2	RFESD	MGST3	TROAP	GRAP2	SNX22	RHCE	GATA1
PBK	RCCD1	LXN	NEK2	APLNR	SLFN14	CISH	PI4K2A
ZHX3	HSPBP1	SLC37A4	TSPAN5	CENPH	GAS2L3	BCL11A	CYTH4
CHAC1	BGLT3	C9orf40	KNSTRN	TMEM120B	FOSB	CTNNAL1	MECR
RELB	IFI16	PRMT6	NCCRP1	POLR3H	ICAM1	RPA3	FAM72B
UBXN10	CDC42EP3	CENPA	RAD54L	PKLR	C3AR1	SNN	BAALC
KCNC4	PCYT1B	PCNX2	STX2	TUBGCP4	ALDH1B1	TSPAN13	H2AC6
LIMA1	HIRA	DPY19L2P2	STAG3L1	WDR4	LINC01366	PSMB9	CLCN2
CR1L	ADA2	PPFIBP1	HLA-DRB1	C3	MIR3142HG	SPRED2	SCAMP5
RAB6B	C1orf109	KIAA1217	CCDC86	BTBD9	ST6GALNAC1	GPR155	SRGAP3
NRP1	NOS1	PPP1R16B	MRM1	PAQR8	SLC25A21	S1PR2	NLN
SEMA6B	HIF3A	SYTL4	RFXAP	SLC26A11	NCF1B	ADGRG1	LRRC20

GPR35	SMIM10	HPGDS	PAQR9	RIMS3	GARIN1A	PLK3	PLD2
DHCR7	LRRC75A	MIPEP	ADAMDEC1	ACRBP	TPSG1	ACVR2A	IL1B
CD70	RRAD	ADAM19	C8orf88	HLX	ARHGEF39	SPATS2L	P2RY6
CCR7	MARCKS	CTH	AQP3	SPTBN4	EBI3	MANSC1	PADI2
SLC16A9	ALDH7A1	KAZALD1	LAMP3	SORBS3	AOC1	TBC1D8	PTGER4
SPIB	ST3GAL5	BOLA3-DT	LRRC25	DPF3	CMKLR1	RRS1	CXCL14
SLC18B1	RASA4B	PDE4B	GIMAP8	FEZ1	STT3A-AS1	NMRAL2P	TMEM150C
NMU	NBPF8	PEAK3	HPDL	LRP11	NR4A3	RGS6	BICDL1
PLXNA2	C5AR1	P2RY13	NFAM1	HIVEP3	B4GAT1	HLA-DOA	BEND6
TVP23A	ACTR3C	OSGIN1	SH2B2	PURPL	HS3ST1	SLC5A4-AS1	A2ML1
INPP4B	RRAS	SRXN1	UTS2R	DLL4	DOCK4	SIGLEC5	ZNF704
PIK3R3	VNN1	TUBB2B	TMEM119	LAMB2	CACNA1H	HDAC9	EDNRB

PARM1	PLA2G4C						

Supplementary Figures

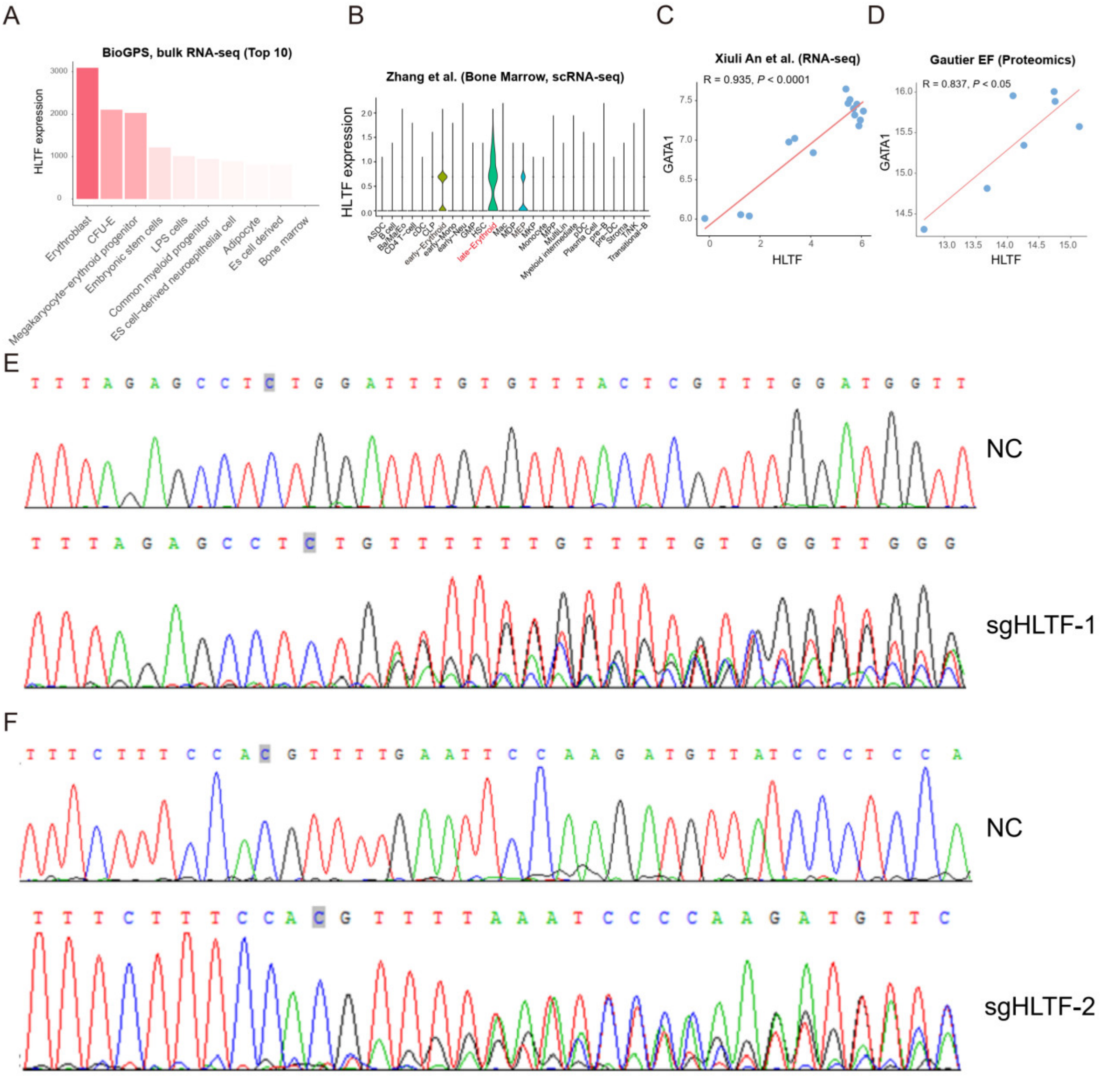

Supplementary Figure S1. HLTF expression during erythroid differentiation and validation of HLTF knockout. (A) HLTF mRNA expression levels across various cell types and tissues from BioGPS bulk RNA-seq data (Top 10). (B) Public single-cell RNA-seq (scRNA-seq) of human bone marrow showing HLTF expression across hematopoietic cell subsets, with marked enrichment in erythroid lineages. (C) Correlation analysis of bulk RNA-

seq data showing a strong positive association between HLTF and GATA1 expression across erythroid differentiation. (D) Correlation plot of HLTF and GATA1 protein expression during erythroid differentiation stages based on proteomics data. (E, F) Sanger sequencing confirming efficient editing of the sgHLTF-1 (E) and sgHLTF-2 (F) by CRISPR/Cas9 in HLTF knockout (KO) erythroid cells. All data are representative of at least three independent experiments. Error bars indicate mean±SD.

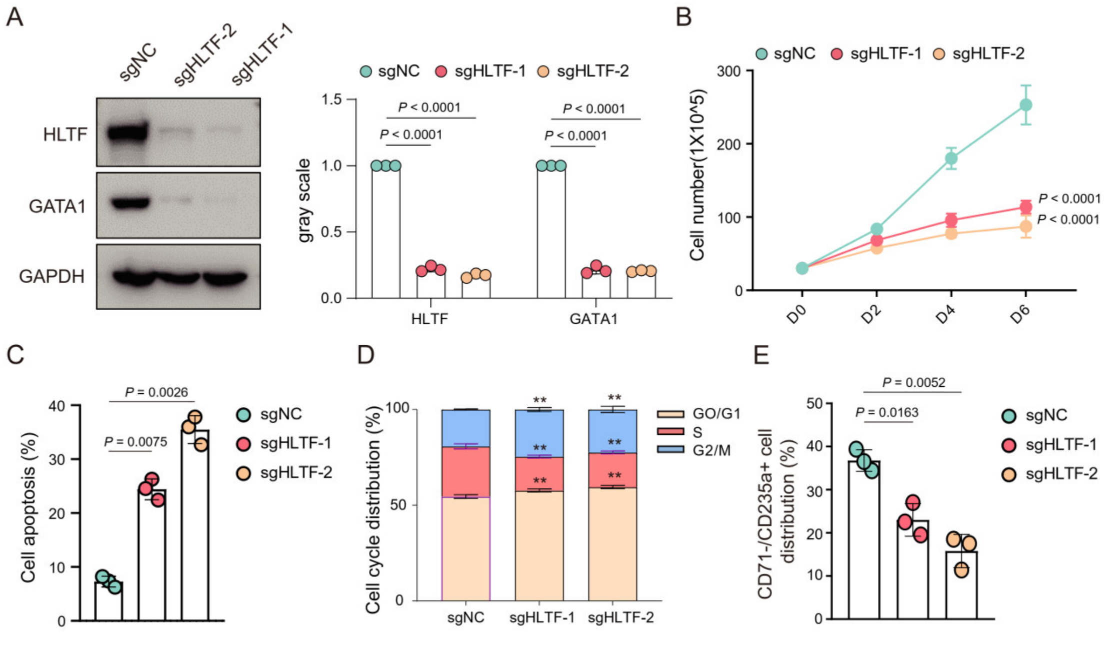

Supplementary Figure S2. Knockout of HLTF impaired erythroid development in HUDEP-2 cells. (A) Western blot analysis of HLTF and GATA1 protein levels in HUDEP-2 cells treated with sgNC, sgHLTF-1, or sgHLTF-2. GAPDH was used as a loading control. The accompanying bar graph shows the quantification of HLTF and GATA1 protein levels. (B) Cell proliferation curves showing cell number over time (D0, D2, D4, D6) for HUDEP-2 cells treated with sgNC, sgHLTF-1, or sgHLTF-2. (C) Quantification of cell apoptosis rates in cells treated with sgNC, sgHLTF-1, or sgHLTF-2. (D) Cell cycle distribution analysis (G0/G1, S,

G2/M phases) in cells treated with sgNC, sgHLTF-1, or sgHLTF-2. (E) Percentage distribution of CD71-/CD235a+ cells in sgNC, sgHLTF-1, and sgHLTF-2 treated HUDEP-2 cells. All data are representative of at least three independent experiments. Error bars indicate mean±SD.

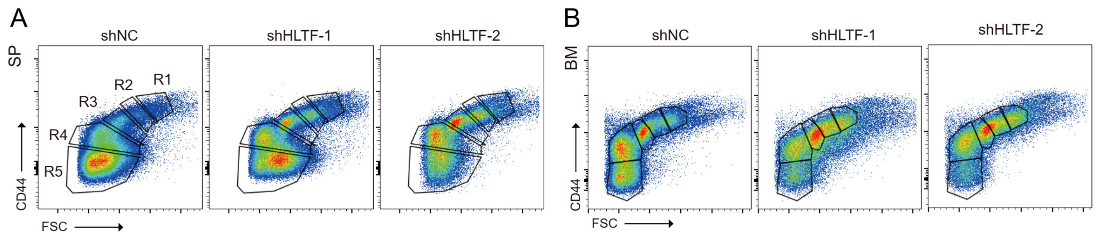

Supplementary Figure S3. Flow cytometry analysis. (A, B) Flow cytometry plots showing CD44 and FSC (Forward Scatter) expression in spleen (SP) cells (A) and bone marrow (BM) cells (B) from mice treated with shNC, shHLTF-1, or shHLTF-2. Distinct cell populations are gated as R1, R2, R3, R4, and R5.

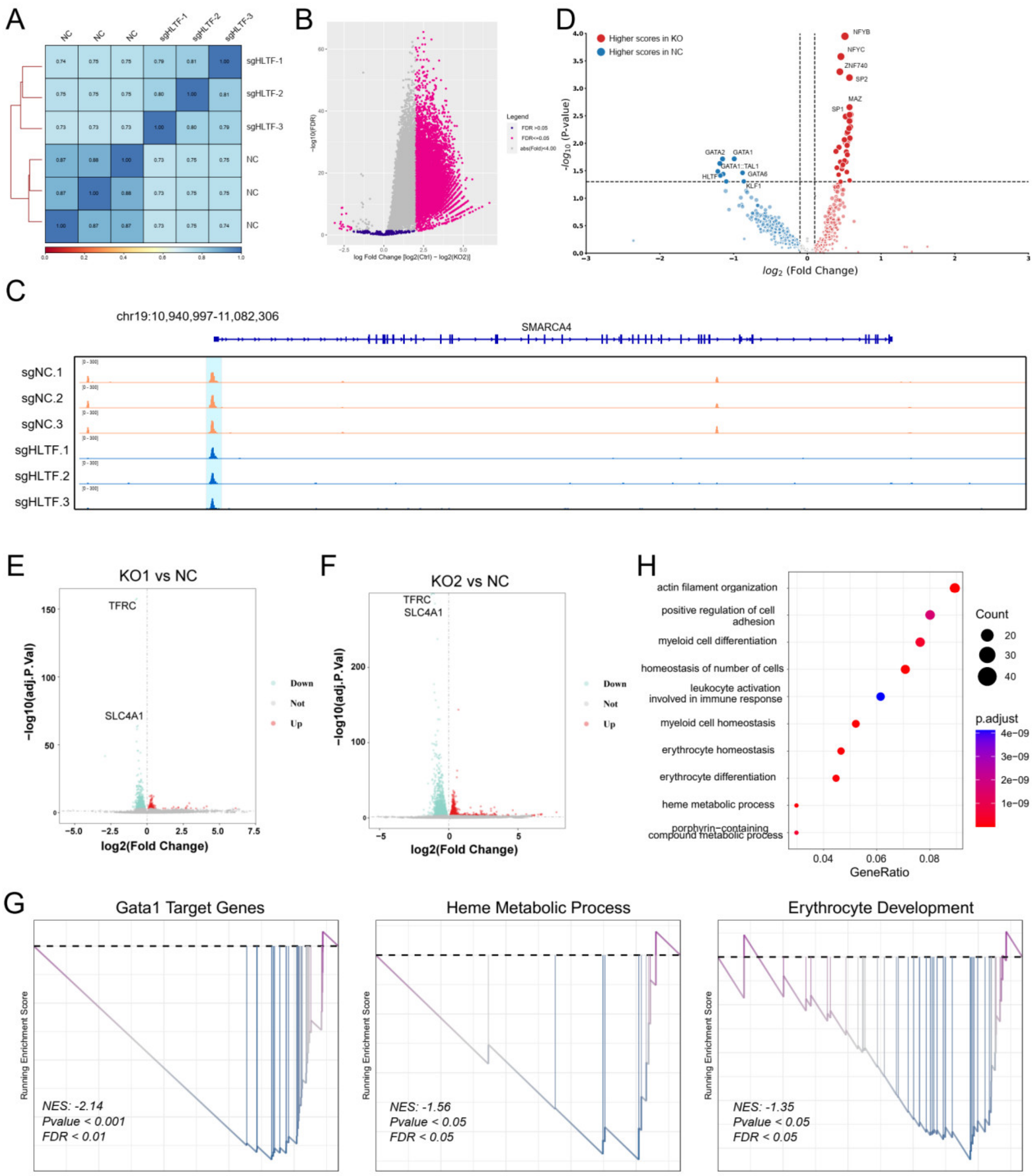

Supplementary Figure S4. Integrated multi-omics analysis confirmed HLTF as a chromatin-associated transcriptional regulator of erythroid gene networks. (A) Heatmap illustrating the correlation between the sgNC and sgHLTF groups. (B) Volcano plot depicting differentially accessible chromatin regions (peaks) between sgHLTF and sgNC conditions. (C) IGV browser tracks at SMARCA4 locus. (D) Footprinting analysis performed on ATAC-seq peaks, displayed as a volcano plot, indicating transcription factors (TFs) with higher binding scores in either HLTF knockout (KO) or control (NC) conditions. (E, F) Volcano plots showing

differentially expressed genes in sgHLTF-1 (E) and sgHLTF-2 (F) groups. (G) Gene Set Enrichment Analysis (GSEA) plots for "Gata1 Target Genes", "Heme Metabolic Process", and "Erythrocyte Development". Each plot shows the running enrichment score (NES), nominal P-value, and False Discovery Rate (FDR), indicating the enrichment of these gene sets. (H) Gene Ontology (GO) enrichment analysis of differentially expressed genes, presented as a bubble chart.

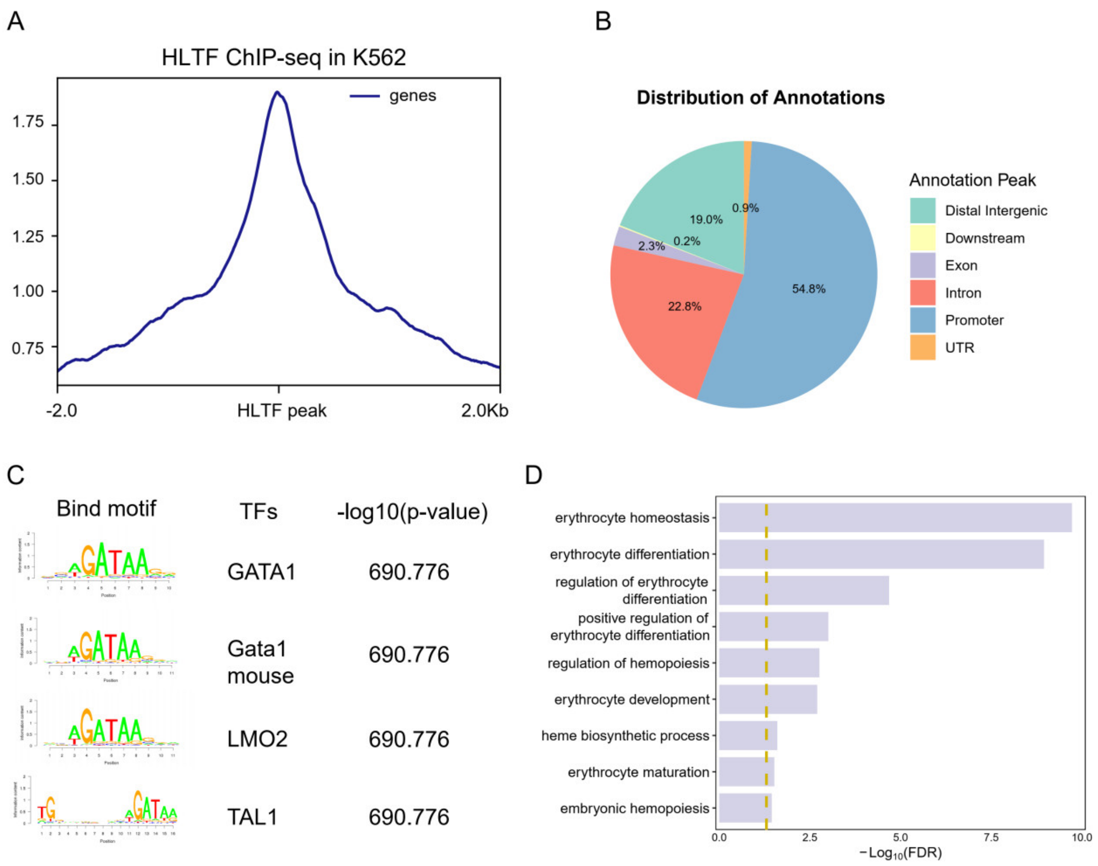

Supplementary Figure S5. ChIP-seq analysis of HLTF in K562 cells. (A) Heatmap displaying HLTF ChIP-seq enrichment in K562 cells around gene regions, centered at HLTF peaks. (B) Pie chart illustrating the genomic distribution of HLTF binding peaks, categorized by their proximity to various genomic annotations (Distal Intergenic, Downstream, Exon,

Intron, Promoter, UTR). (C) Enriched binding motifs identified from HLTF ChIP-seq data, along with their corresponding transcription factors (TFs) such as GATA1, LMO2, and TAL1, and their respective -log10(p-values). (D) Gene Ontology (GO) enrichment analysis of genes associated with HLTF binding, presented as a bubble chart.

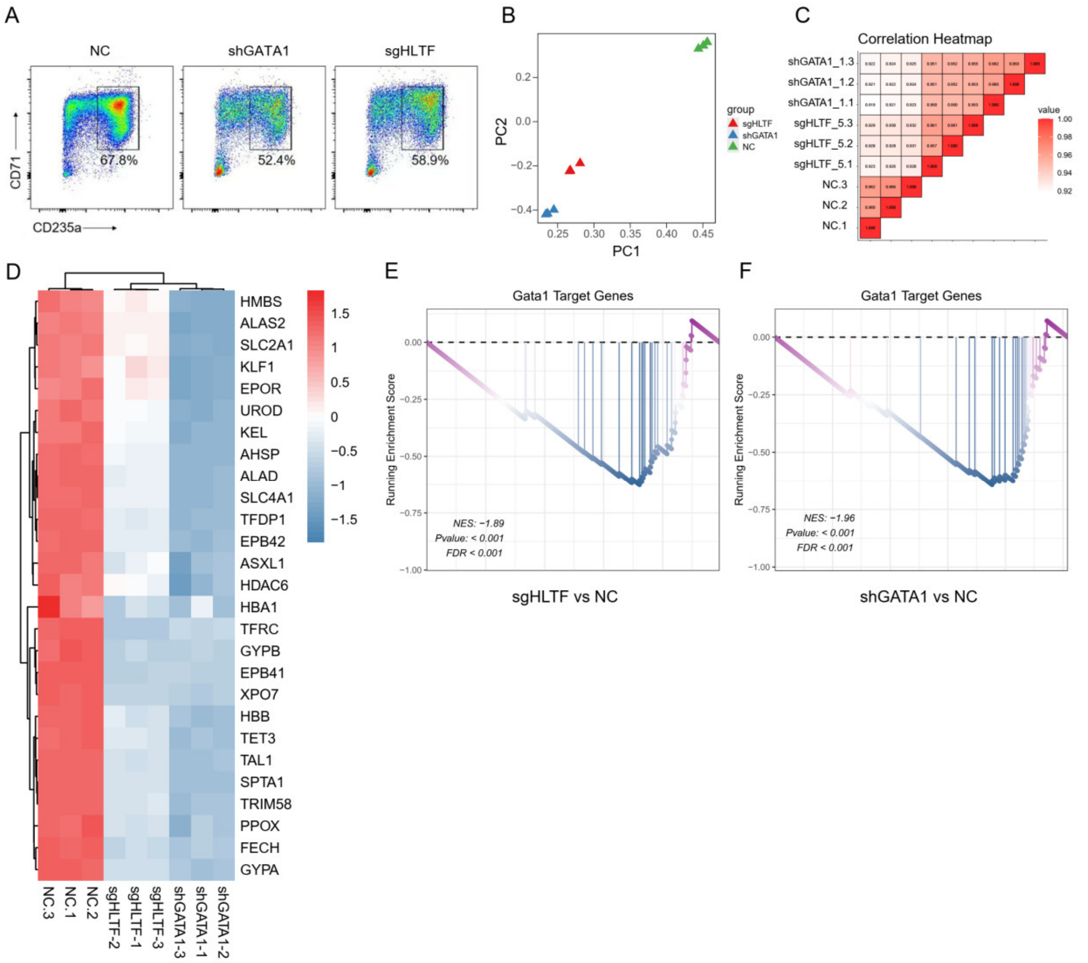

Supplementary Figure S6. Transcriptomic analysis of HLTF- and GATA1-deficient erythroid cells. (A) Flow-cytometric sorting of $CD71^{+}CD235a^{+}$ erythroid cells transduced with negative control (NC), shGATA1, or sgHLTF for bulk RNA-seq analysis. (B) Principal component analysis (PCA) of RNA-seq data. (C) Correlation analysis illustrating high within-group reproducibility and distinct transcriptomic separation between HLTF- and GATA1-

deficient cells. (D) Heatmaps showing the relative expression of representative erythroid-associated genes across NC, sgHLTF, and shGATA1 groups. (E, F) Gene Set Enrichment Analysis (GSEA) of GATA1 target genes in HLTF-deficient (E) and GATA1-deficient (F) cells.

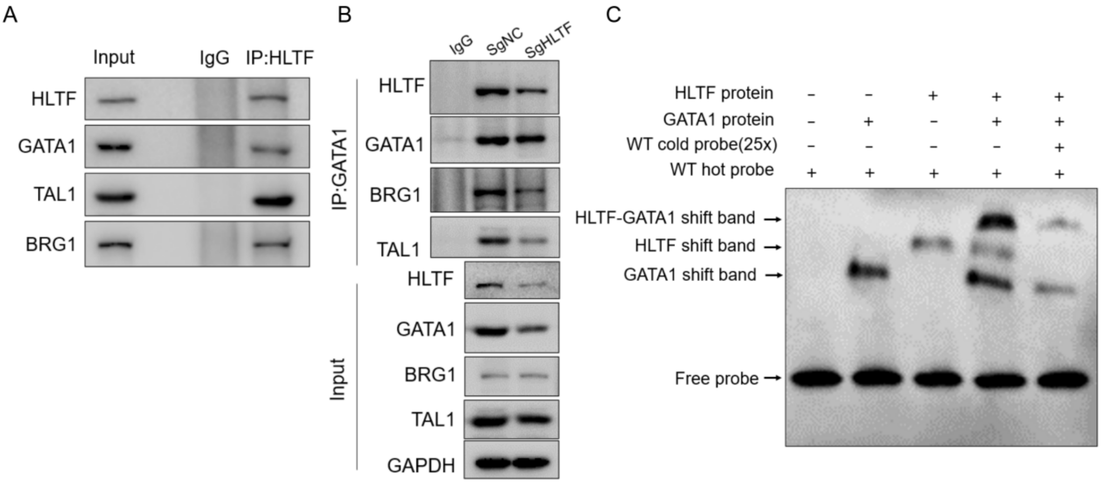

Supplementary Figure S7. HLTF participates in the TAL1–GATA1–BRG1 complex. (A) Co-immunoprecipitation (Co-IP) assays showing that HLTF interacts with GATA1, TAL1, and BRG1 in CD34-derived erythroid cells. (B) HLTF knockdown weakened the interaction between GATA1 and its cofactors TAL1 and BRG1. (C) Electrophoretic mobility shift assay (EMSA) using purified HLTF and GATA1 proteins and a biotin-labeled probe containing the GATA1 binding site.

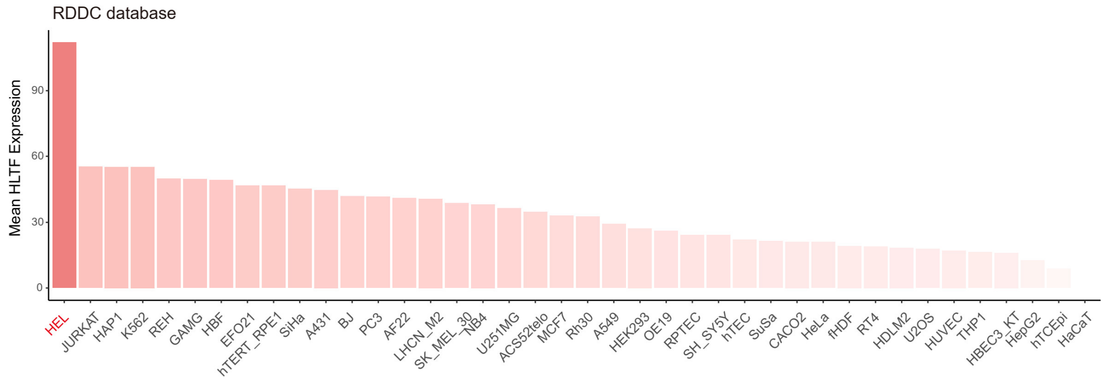

Supplementary Figure S8. HLTF expression level across a variety of cells.

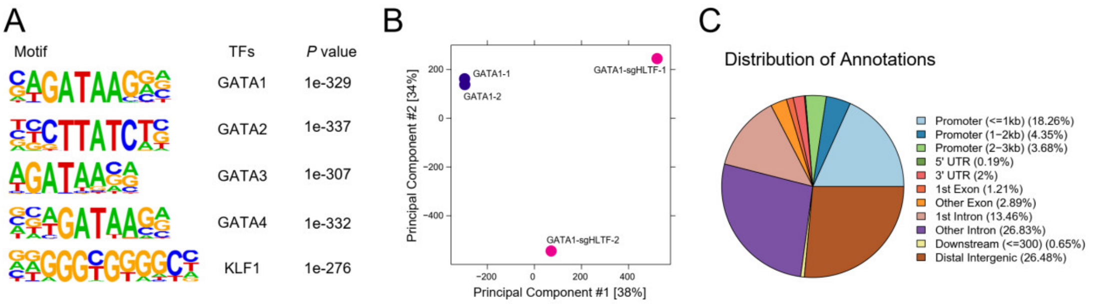

Supplementary Figure S9. Chromatin accessibility and GATA1 motif enrichment in HLTF-deficient PV cells. (A) Motif enrichment analysis of differentially accessible regions in ATAC-seq. (B) Principal Component Analysis (PCA) plot of GATA1 CUT&Tag following HLTF knock out. (C) Genomic distribution of downregulated GATA1-bound regions.
